# Emergence and phenotypic characterization of the global SARS-CoV-2 C.1.2 lineage

**DOI:** 10.1038/s41467-022-29579-9

**Published:** 2022-04-08

**Authors:** Cathrine Scheepers, Josie Everatt, Daniel G. Amoako, Houriiyah Tegally, Constantinos Kurt Wibmer, Anele Mnguni, Arshad Ismail, Boitshoko Mahlangu, Bronwen E. Lambson, Darren P. Martin, Eduan Wilkinson, James Emmanuel San, Jennifer Giandhari, Nelia Manamela, Noxolo Ntuli, Prudence Kgagudi, Sandile Cele, Simone I. Richardson, Sureshnee Pillay, Thabo Mohale, Upasana Ramphal, Yeshnee Naidoo, Zamantungwa T. Khumalo, Gaurav Kwatra, Glenda Gray, Linda-Gail Bekker, Shabir A. Madhi, Vicky Baillie, Wesley C. Van Voorhis, Florette K. Treurnicht, Marietjie Venter, Koleka Mlisana, Nicole Wolter, Alex Sigal, Carolyn Williamson, Nei-yuan Hsiao, Nokukhanya Msomi, Tongai Maponga, Wolfgang Preiser, Zinhle Makatini, Richard Lessells, Penny L. Moore, Tulio de Oliveira, Anne von Gottberg, Jinal N. Bhiman

**Affiliations:** 1 National Institute for Communicable Diseases (NICD) of the National Health Laboratory Service (NHLS), 2131 Johannesburg, South Africa; 2 SA MRC Antibody Immunity Research Unit, School of Pathology, Faculty of Health Sciences, University of the Witwatersrand, 2132 Johannesburg, South Africa; 3 KwaZulu-Natal Research Innovation and Sequencing Platform (KRISP), Nelson R Mandela School of Medicine, University of KwaZulu-Natal, 4001 Durban, South Africa; 4 Centre for Epidemic Response and Innovation (CERI), School of Data Science and Computational Thinking, Stellenbosch University, 7600 Stellenbosch, South Africa; 5 Institute of Infectious Disease and Molecular Medicine, University of Cape Town, 7925 Cape Town, South Africa; 6 Africa Health Research Institute, 4001 Durban, South Africa; 7 School of Laboratory Medicine and Medical Sciences, University of KwaZulu-Natal, 4041 Durban, South Africa; 8 Department of Veterinary Tropical Diseases, Faculty of Veterinary Science, University of Pretoria, 0110 Onderstepoort, South Africa; 9 South African Medical Research Council Vaccines and Infectious Diseases Analytics Research Unit, University of the Witwatersrand, 2000 Johannesburg, South Africa; 10 Department of Clinical Microbiology, Christian Medical College, Vellore, 632004 India; 11 South African Medical Research Council, 7505 Cape Town, South Africa; 12 Desmond Tutu HIV Centre, 7925 Cape Town, South Africa; 13 Center for Emerging and Re-emerging Infectious Diseases (CERID), University of Washington, Washington, 98195 USA; 14 National Health Laboratory Service (NHLS), 2131 Johannesburg, South Africa; 15 Faculty of Health Sciences, University of the Witwatersrand, 2193 Johannesburg, South Africa; 16 Zoonotic Arbo and Respiratory Virus Research Programme, Department of Medical Virology, Faculty of Health, University of Pretoria, 0031 Gezina, South Africa; 17 Clinical Microbiology and Infectious Diseases Unit, Faulty of Health Sciences, University of Witwatersrand, 2132 Johannesburg, South Africa; 18 Max Planck Institute for Infection Biology, 10117 Berlin, Germany; 19 Wellcome Centre for Infectious Diseases Research in Africa (CIDRI-Africa), 7925 Cape Town, South Africa; 20 Discipline of Virology, University of KwaZulu-Natal, 4041 Durban, South Africa; 21 Division of Medical Virology, Faculty of Medicine and Health Sciences, Stellenbosch University, Tygerberg, 7602 Cape Town, South Africa; 22 Centre for the AIDS Programme of Research in South Africa (CAPRISA), 4013 KwaZulu-Natal, South Africa; 23 Faculty of Health Sciences, University of Cape Town, 7935 Cape Town, South Africa

**Keywords:** SARS-CoV-2, Viral infection, Epidemiology

## Abstract

Global genomic surveillance of SARS-CoV-2 has identified variants associated with increased transmissibility, neutralization resistance and disease severity. Here we report the emergence of the PANGO lineage C.1.2, detected at low prevalence in South Africa and eleven other countries. The initial C.1.2 detection is associated with a high substitution rate, and includes changes within the spike protein that have been associated with increased transmissibility or reduced neutralization sensitivity in SARS-CoV-2 variants of concern or variants of interest. Like Beta and Delta, C.1.2 shows significantly reduced neutralization sensitivity to plasma from vaccinees and individuals infected with the ancestral D614G virus. In contrast, convalescent donors infected with either Beta or Delta show high plasma neutralization against C.1.2. These functional data suggest that vaccine efficacy against C.1.2 will be equivalent to Beta and Delta, and that prior infection with either Beta or Delta will likely offer protection against C.1.2.

## Abstract (web summary)

The SARS-CoV-2 PANGO lineage C.1.2 has been under monitoring by global health authorities as it has spread worldwide. Here, Bhiman and colleagues characterise the emergence of the lineage, and its neutralisation sensitivity using data from vaccinees and previously infected individuals.

## Introduction

Almost two years into the COVID-19 pandemic, SARS-CoV-2 remains a global public health concern with ongoing waves of infection resulting in the selection of variants with novel constellations of mutations within the viral genome^1–3^. Some variants accumulate mutations within the spike region that result in increased transmissibility and/or immune evasion, making them of increased public health importance^1–3^. Depending on their clinical and epidemiological profiles, these are either designated as variants of interest (VOI) or variants of concern (VOC) (www.who.int), and ongoing genomic surveillance is essential for early detection of such variants. At the time of writing there were four VOCs (Alpha, Beta, Gamma, and Delta) and five VOIs (Eta, Iota, Kappa, Lambda, and Mu) in circulation globally (www.who.int, Accessed September 21, 2021). Alpha, Beta, and Delta have had the most impact in terms of transmission and immune evasion, with Delta rapidly displacing other variants to dominate globally and in South Africa^4^.

Ongoing genomic surveillance by the Network for Genomic Surveillance in South Africa (NGS-SA)^5^ detected an increase in sequences assigned to C.1 during the country’s third wave of SARS-CoV-2 infections from March 2021. This was unexpected since C.1, first identified in South Africa^6,7^, was last detected in January 2021. Upon comparison of the mutational profiles between the new and older C.1 sequences (which only contain the D614G spike mutation), it was clear that the new sequences had mutated substantially. C.1 had minimal spread globally, but was detected in Mozambique where it was found to have accumulated additional mutations, resulting in the PANGO lineage C.1.1^7^. The new C.1-like sequences discovered from March 2021 were, however, very distinct from C.1.1, and were subsequently assigned to the PANGO lineage C.1.2. Here, we describe the emergence and phenotypic characterization of the C.1.2 lineage, which is distinct from the now globally dominant Omicron VOC^8^. The C.1.2 lineage shares many mutations with other VOC/VOIs but did not become a dominant lineage in South Africa or globally, likely due to its sensitivity to neutralization by convalescent plasma from both Beta and Delta infected individuals.

## Results

### Emergence and global detection of the C.1.2 lineage

As of data deposited on September 10, 2021 we identified 166 sequences that match the C.1.2 lineage (available on GISAID^9^ (www.gisaid.org), the global reference database for SARS-CoV-2 viral genomes, and listed in Supplementary Tables 1 and 2). While these 166 sequences make up the C.1.2 dataset used in this analysis, at the time of submission (September 22, 2021) a further 11 C.1.2 sequences had been deposited in GISAID, including detection in the United States of America. The majority of the 166 sequences (*n* = 146, including ten from vaccine breakthrough cases (Supplementary Table 1)) are from South Africa. The remaining sequences were detected in Botswana, China, the Democratic Republic of the Congo (DRC), Eswatini, England, Mauritius, New Zealand, Portugal, Switzerland, and Zimbabwe, with at least 12/20 cases having a travel history (Supplementary Fig. 1a and Supplementary Table 2). Provincial detection of C.1.2 mirrored the depth of sequencing across South Africa (Supplementary Fig. 1b–d), suggesting that the observed numbers may be an underrepresentation of the spread and frequency of this variant within South Africa and globally. We observed increased detection rates of C.1.2 in May, June and July (Supplementary Fig. 1e). Although the C.1.2 detection rate decreased in August, very few sequences have been obtained for the end of the month based on data submitted to GISAID as of September 21, 2021 (Supplementary Fig. 1d). Nevertheless, these rates are similar to the increases seen at the start of Beta and Delta detection in South Africa, though notably a sudden large increase in C.1.2 has not yet occurred (Supplementary Fig. 1e). Spatiotemporal phylogeographic analysis predicts the root of the C.1.2 lineage around mid-July 2020 (95% highest posterior density ranging from May to October 2020) in Gauteng or surrounding provinces (Supplementary Fig. 1f). Onward spread from Gauteng to neighboring provinces is inferred to have occurred in early 2021, while introduction to more distant provinces occurred from the end of May 2021.

### C.1.2 lineage divergence and mutational profile

**Fig. 1:**
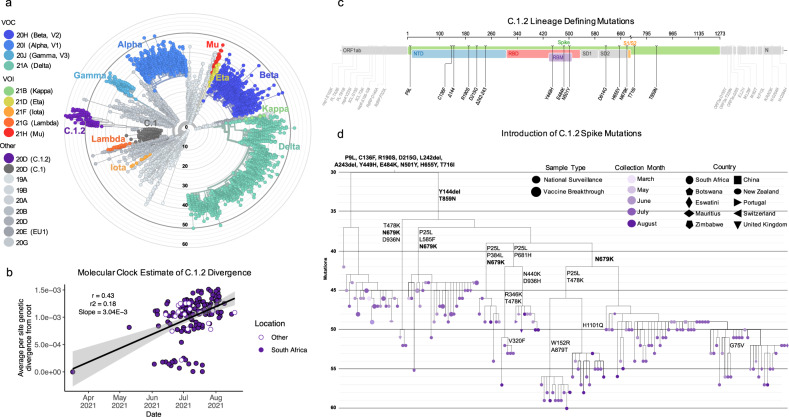
C.1.2 a highly mutated SARS-CoV-2 variant. **a** Phylogenetic tree of 6,192 global sequences (1,991 sequences from South Africa), including variants of concern (VOC), variants of interest (VOI), and the C.1.2 lineage, colored by Nextstrain clade (shown in the key; VOCs and VOIs are in color, other clades in greyscale) and scaled by divergence (number of mutations). The C.1.2 lineage (purple) forms a distinct, highly mutated cluster within clade 20D. **b** Regression of root-to-tip genetic distances against sampling dates for sequences belonging to lineage C.1.2 sampled either in South Africa (solid purple) or in other countries (white) indicating that the C.1.2 sequences evolved in a clock-like manner (correlation coefficient = 0.43, *R*^2^ = 0.18). The regression gradient is an estimate of the rate of sequence evolution, which is 3.04 × 10^−3^ nucleotide substitutions/site/year. Regression lines are shown with error buffers (shaded area) representing 90% confidence intervals. **c** Full genome representation of C.1.2 showing lineage defining mutations (seen in ≥50% of C.1.2 assigned sequences), with those in the spike (green) colored according to functional regions, including the N-terminal domain (NTD, blue), receptor binding domain (RBD, red), receptor binding motif (RBM, purple), subdomain 1 and 2 (SD1 or SD2, gray) and the cleavage site (S1/S2, yellow). Figure generated by covdb.stanford.edu. **d** A phylogenetic tree highlighting the introduction of spike mutations in the different sub-clades of the C.1.2 lineage. The tree is colored by month of collection, with tip symbols indicating country of collection, as indicated in the key (Figure generated from a Nextstrain build of global C.1.2 sequences with ≥90% coverage). Mutations in bold represent those observed in ≥50% of the C.1.2 sequences.

C.1.2 is highly mutated, even when compared to circulating VOI/VOCs (Fig. 1a). The emergence of the Alpha, Beta, Delta, and Gamma variants were associated with short periods of notably increased evolution compared to the overall SARS-CoV-2 evolutionary rate^10^. The same is observed for C.1.2, which has an approximate evolutionary rate of 3.04 × 10^−3^ nucleotide changes/site/year (Fig. 1b). The short periods of increased evolution associated with the emergence of this and other new lineages are indicative of the accumulation of mutations having occurred during discrete epidemiological events, such as virus–host co-evolution within individuals with prolonged viral infections^11,12^, or genetic recombination between two distantly related viral variants co-infecting the same individual. Although C.1.2 shares multiple mutations with the Alpha, Beta, and Delta variants, we were unable to find evidence for recombination events between these VOCs or C.1 within C.1.2 viruses (Supplementary Fig. 2c). Both the absence of evidence of recombination, and the phylogenetic placement of C.1.2 relative to the other VOCs, support the hypothesis that the mutations in C.1.2 likely occurred as a result of convergent evolution. Diversifying selective pressures are an important contributor to the maintenance of mutations, which confer evolutionary advantages to the virus, and one such example where this may occur is in the context of prolonged infection within an individual^11,12^. However, as global coverage of vaccines using the spike originally identified in Wuhan increases, the impact of suboptimal vaccine-elicited responses is likely to also contribute to selection of these types of viral mutations.

C.1.2 has accumulated additional mutations within the *ORF1ab*, *S*, *ORF3a*, *ORF9b*, *E*, *M*, and *N* genes compared to its C.1 ancestor (Fig. 1c). C.1.2 has, on average, 30 amino acid substitutions across its entire genome, similar to the frequency observed in Delta (average: 28) (Supplementary Fig. 2a). On average, 13 of these amino acid substitutions were observed within the spike protein, notably higher than Delta (average: 7) but similar to Gamma (average: 12) (Supplementary Fig. 2a). The spike protein substitutions observed in C.1.2 include five within the N-terminal domain (NTD: C136F, Y144del, R190S, D215G, and 242-243del or 243-244del (either deletion results in the same amino acid sequence)), three within the receptor binding motif (RBM: Y449H, E484K, and N501Y) and three adjacent to the furin cleavage site (H655Y, N679K, and T716I) (Fig. 1c). Though these substitutions, along with P9L and T859N, occur in the majority of C.1.2 viruses, there is additional variation within the spike region of this lineage (Supplementary Fig. 2b, c). Additional substitutions include P25L (in ~43% of viruses) and W152R (in ~7%) in the NTD, T478K (~17%) in the RBM, L585F (~17%) in S1, P681H (~8%) adjacent to the furin cleavage site, A879T (~7%), D936H (~5%), and H1101Q (~8%) in S2, with additional amino acid changes detected in less than 5% of viruses (Supplementary Fig. 2b). Though the prevalence of some of the mutations causing these substitutions may be underestimated due to lack of sequencing coverage in parts of the *S* gene (Supplementary Fig. 2c), their presence, particularly in the most recent samples, suggests ongoing intra-lineage evolution. The majority of these substitutions (P9L, C136F, R190S, D215G, L242del, A243del, Y449H, E484K, N501Y, H655Y, and T716I), however, appeared simultaneously, further supporting a single, prolonged infection giving rise to this lineage (Fig. 1d).

### Impact of C.1.2 lineage spike mutations on phenotype

**Fig. 2:**
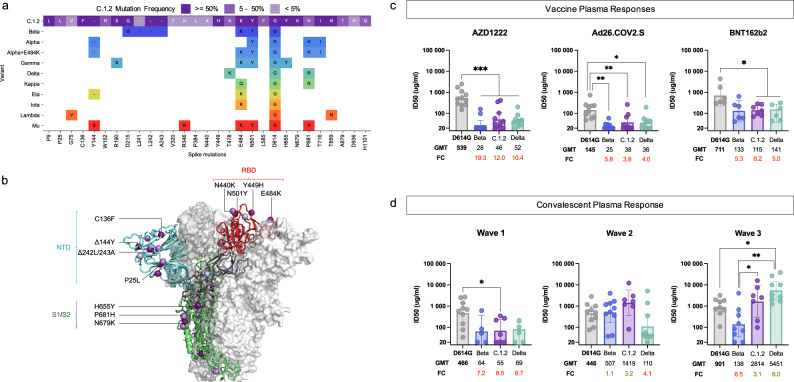
Shared spike mutation results in similar immune responses. **a** Visualization of C.1.2 spike mutations, highlighting those shared with VOCs and VOIs (colored by the Nextstrain clade). All C.1.2 mutations are shown, and colored according to prevalence within the C.1.2 sequences (shown in key). For VOCs and VOIs only mutations present in at least 50% of sequences are shown (as determined by frequency information at outbreak.info, accessed September 13, 2021). **b** Schematic showing C.1.2 mutations on the RBD-down conformation of SARS-CoV-2 spike, with domains of a single protomer shown in cartoon view and colored cyan (N-terminal domain, NTD), red (C-terminal domain/receptor binding domain, CTD/RBD), gray (subdomain 1 and 2, SD1 and SD2), and green (S2). The adjacent protomers are shown in translucent surface view and colored shades of gray. Lineage-defining mutations (found in >50% of sequences) are colored dark purple, with additional mutations (present in <50% of sequences) colored light purple. Key mutations known/predicted to influence neutralization sensitivity (C136F and P25L, Δ144Y, Δ242L/243A, and E484K), or furin cleavage (H655Y and N679K) are indicated. Image was created using the PyMOL molecular graphics program. **c**, **d** Neutralization activity of biologically independent plasma samples taken from donors previously vaccinated with either AZD1222 (*N* = 11), Ad26.COV.2.S (*N* = 10 for D614G, Beta and Delta and *N* = 9 for C.1.2) or BNT162b2 (*N* = 7 for C.1.2 and *N* = 6 for D614G, Beta, and Delta) (**c**) and patients previously infected during the first (*N* = 10 for D614G, *N* = 7 for C.1.2 and *N* = 5 for Beta and Delta), second (*N* = 10 for D614G, Beta, and Delta and *N* = 7 for C.1.2) or third (*N* = 9 for D614G, Beta, and Delta and *N* = 7 for C.1.2) waves in South Africa (**d**) against the wild-type (D614G), Beta, Delta, and C.1.2 variants. Bar graphs represent the geometric mean titer (GMT) for each group with the error bars representing the 95% confidence intervals, dots represent individual sample titers. Statistical significance based on the Wilcoxon two-tailed matched-pairs signed rank test are shown above graphs. *p*-values are denoted with “*” symbols: *p < 0.05, **p < 0.01, and ***p < 0.001. GMT and fold-change (FC) differences relative to D614G are given below the graph, with red representing decreased titer and green representing increased titer. No adjustments for multiple testing were made.

Of the 28 spike protein substitutions identified in C.1.2, 14 (50%) have been previously identified in other VOIs and VOCs, including mutations associated with increased transmissibility and/or neutralization escape (Fig. 2a). These substitutions include D614G, common to most circulating SARS-CoV-2 variants and associated with increased viral fitness^13^, and E484K and N501Y in the RBD, which are shared with multiple VOCs and VOIs and associated with reduced antibody responses^14,15^. C.1.2 contains another two RBD mutations not seen in other VOIs or VOCs: N440K and Y449H, which co-localize on the same outer face of the RBD (Fig. 2b). While these two changes are not characteristic of current VOCs/VOIs, they have been associated with escape from certain class 3 neutralizing antibodies^16^. The Y144del and 242-244del cause shifts in the immunodominant N3 and N5 loops of the NTD (blue, Fig. 2b) in the Alpha and Beta variants, respectively, altering antigenicity of the region^14^. Recurrent NTD deletions have also previously been associated with prolonged SARS-CoV-2 infection^17^. Furthermore, the C136F mutation abolishes a disulfide bond within the N1 loop of NTD, and in combination with P25L likely contributes to immune escape by conformationally liberating the entire N-terminus of the NTD. The P9L within the signal peptide is predicted to increase translation. Amino acid substitutions close to the furin cleavage site, including H655Y and P681R/H, have been shown to increase S1/S2 cleavage efficiency, and have also been observed in other VOCs and VOIs, such as Alpha, Delta, Gamma, Mu, and Kappa^18–20^ (S1/S2 region in Fig. 2b). In the C.1.2 lineage, N679K and P681H are mutually exclusive (with N679K predominating, Fig. 1d), which suggests that they may perform a similar role by increasing the local, relative positive charge of the furin cleavage site to potentially improve the furin cleavage efficiency. Similar substitutions have been seen within Gamma^21^. Finally, T859N has been detected in Lambda as well as multiple non-VOC/VOI lineages and is predicted to affect spike stability in a similar way as the D614G substitution. The identification of convergent evolution between C.1.2 and the presently designated VOIs and VOCs suggests that this lineage may also share concerning phenotypic properties with these VOIs and VOCs.

Plasma neutralizing activity from donors who received the ChAdOx1 nCoV-19 (AZD1222), Jansen/Johnson and Johnson (Ad26.COV2.S) or Pfizer/BioNTech (BNT162b2) vaccines, showed 3 to 12-fold reduction in antibody titers for C.1.2 compared to the original D614G variant (containing only the D614G spike mutation). Despite this, no statistically significant difference in the reduction in titers against Beta (5 to 19-fold) or Delta (4 to 10-fold) was observed (Fig. 2c and Supplementary Fig. 3a). Similarly, convalescent plasma from donors infected with the D614G variant that dominated the first wave of infections in South Africa^6^ showed reduced responses to Beta (geometric mean titer, GMT of 64), C.1.2 (GMT: 55) and Delta (GMT: 69) (Fig. 2d, left panel and Supplementary Fig. 3b). Convalescent plasma from donors infected during the second wave of infections in South Africa (dominated by Beta), showed reduced sensitivity against Delta (GMT: 110) but high neutralizing activity against C.1.2 (GMT: 1419). This may be attributed to the shared E484K and N501Y in the RBD of the Beta and C.1.2 variants, which are absent in Delta (Fig. 2d, middle panel and Supplementary Fig. 3a, middle panel). In donors infected during South Africa’s third wave of Delta-dominated infections^4^, neutralizing activity was reduced against D614G and Beta, but relatively high titers were observed against C.1.2 (GMT: 2814) (Fig. 2d, right panel and Supplementary Fig. 3a, right panel). High neutralization titers against D614G, C.1.2, and Delta are likely a result of higher viral loads associated with Delta infections^22^, while reduced titers against Beta may be a result of the K417N substitution, which escapes a predominant antibody class^14^, and is not present in Delta or C.1.2. Antibody dependent cellular cytotoxicity (ADCC) in both vaccinees and wave one convalescent donors was significantly reduced against C.1.2 relative to D614G. (Fig. 2c and Supplementary Fig. 3b). In contrast, as with neutralization, ADCC responses against C.1.2 in convalescent plasma from individuals infected in waves two and three, were largely preserved (Supplementary Fig. 3b).

## Discussion

Overall these results show that, as with Beta and Delta, vaccine-induced antibody responses show reduced activity against the C.1.2 variant. However, prior infection with either Beta or Delta will likely confer some protection against C.1.2. Cross-reactivity between C.1.2 and Beta/Delta could be a result of shared mutations between these viruses. Though C.1.2 is present in South Africa and globally, we have not yet seen exponential expansion of this lineage as was observed prior to local Beta and Delta dominance^4^. Increased population immune protection through cross-reactive antibodies induced by prior Beta or Delta infections may have limited the spread of C.1.2; the effect of waning immunity may alter this outcome in future resurgences.

Standard strict molecular clock estimates were used to determine the evolutionary rate of C.1.2. While this approximation allowed comparison between this and other VOCs, it may not accurately determine evolutionary rates involving short bursts of substitution, and so this should be considered a preliminary estimate. Nevertheless, we observed similar evolutionary rates in C.1.2 to those of other VOCs. The landscape of the SARS-CoV-2 pandemic is changing at a fast pace and it is almost impossible to be up to date with changes in numbers and types of variants in real-time. Thus at the time of writing we locked our analysis dataset to the 166 sequences that were in GISAID as of September 10, 2021. The number of sequences obtained is strongly influenced by testing capacity, which is generally severely restricted during resurgence periods and may not be a true reflection of the dataset. Furthermore, the recombination analysis was focused on only a small representative selection of the dominant lineages that were circulating in South Africa during 2021. This focus was necessary because the number of tests needed to detect recombination in any dataset scales as a cube of the number of sequences analysed, and, therefore, so does the severity of the multiple testing correction (in this case Bonferroni correction) needed to defend against the detection of false positive recombination signals. Counter-intuitively, by limiting the number of recombination tests to just those between sequences that are representative of the diversity within the main lineages, the recombination analysis maximized our power to detect recombination events that may have involved the C.1.2 lineage. Despite these limitations we have provided insights into the detection, evolution and phenotypic characteristics of C.1.2 within South Africa and globally.

## Methods

### Samples and ethics approvals

SARS-CoV-2 genomic surveillance: As part of monitoring viral evolution by the Network for Genomics Surveillance of South Africa (NGS-SA)^5^, seven sequencing hubs receive randomly selected samples for sequencing every week according to approved protocols at each site. These samples include remnant nucleic acid extracts or remnant nasopharyngeal and oropharyngeal swab samples from routine diagnostic SARS-CoV-2 PCR testing, from public and private laboratories in South Africa. Permission was obtained for associated metadata for the samples including date and location (district and province) of sampling, and sex and age of the patients to offer additional insights about the epidemiology of the infection caused by the virus. The project was approved by the University of the Witwatersrand Human Research Ethics Committee (HREC) (ref. M180832, M210159, and M210752), University of KwaZulu–Natal Biomedical Research Ethics Committee (ref. BREC/00001510/2020), Stellenbosch University HREC (ref. N20/04/008_COVID19) and the University of Cape Town HREC (ref. 383/2020) and the University of Pretoria, Faculty of Health human ethics committee, (ref H101-2017). Individual participant consent was not required for the genomic surveillance as this requirement was waived by the HRECs. This manuscript represents genomic data generated by the NGS-SA consortium and the ethics clearance for performing this genomic surveillance was given with the need for informed consent waived as mentioned above. This study resulted from our routine genomic surveillance as reported in our previous articles, namely Tegally et al.^2^, Tegally et al.^6^, and Viana et al.^8^. Permission to access, publish, and upload the sequence data and accompanying metadata to GISIAD was obtained from each of the HRECs listed above.

Hospitalized Steve Biko Cohort: This study has been given ethics approval by the University of Pretoria, Human Research Ethics Committee (Medical) (247/2020). Serum samples were obtained (longitudinally) from hospitalized patients with PCR-confirmed SARS-CoV-2 infection, known HIV status and aged ≥18 years. These samples have previously been used to assess antibody responses to the D614G (*N* = 10 Wave 1; *N* = 9 Wave 3) and Beta (*N* = 5 Wave 1; *N* = 9 Wave 3) variants^14^, a subset of these were used to measure wave 1 and wave 3 immune responses to Delta (*N* = 5 Wave 1; *N* = 9 Wave 3) and C.1.2 (*N* = 7 Waves 1 and 3) using the pseudovirus neutralization assay and ADCC activity against D614G (*N* = 10 Wave 1; *N* = 9 Wave 3) and C.1.2 (*N* = 10 Wave 1; *N* = 9 Wave 3). All sample sizes represent biologically independent samples (Supplementary Table 3). These samples had a median of 3 days post-PCR test.

Groote Schuur Hospital Cohort: Plasma samples were obtained from hospitalized COVID-19 patients with moderate disease admitted to Groote Schuur Hospital cohort, Cape Town from 30 December 2020 to 15 January 2021 during the second wave in South Africa. All patients were aged ≥18 years and were HIV negative (Supplementary Table 3). This study received ethics approval from the Human Research Ethics Committee of the Faculty of Health Sciences, University of Cape Town (R021/2020). Neutralization activity has previously been assessed for this cohort against the D614G (*N* = 10) and Beta (*N* = 10) variants^23^, a subset of these were used to measure wave 2 antibody responses against Delta (*N* = 10) and C.1.2 (*N* = 7) using the pseudovirus neutralization assay and ADCC (*N* = 10) activity against D614G and C.1.2.

ChAdOx1 nCOV-19 (AZD1222) Vaccinees: samples from donors vaccinated with the ChAdOx1 nCOV-19 (AZD1222) vaccine were previously assessed for neutralization activity against the D614G (*N* = 11) and Beta variants (*N* = 11)^24^. This study received ethics approval from the Pan African Clinical Trials Registry (PACTR202006922165132) as well as the South Africa Health Products Regulatory Authority (SAHPRA: 20200407). A subset of eleven of these samples were used to test neutralization activity against Delta (*N* = 11) and C.1.2 (*N* = 11) using the pseudovirus neutralization assay and ADCC (*N* = 9) activity against D614G and C.1.2. All samples represent biologically independent samples (Supplementary Table 4).

Janssen/Johnson and Johnson (Ad26.COV2.S) Vaccinees: samples from healthy donors vaccinated with the Janssen/Johnson and Johnson (Ad26.COV2.S) vaccine during the Sisonke Trial were obtained 2 months after vaccination and used for the pseudovirus neutralization against D614G (*N* = 10), Beta (*N* = 10), Delta (*N* = 10), and C.1.2 (*N* = 9) and ADCC activity against D614G (*N* = 15) and C.1.2 (*N* = 15). All samples represent biologically independent samples (Supplementary Table 4). Ethics approval for the use of these samples were obtained from the Human Research Ethics Committee of the Faculty of Health Sciences, University of the Witwatersrand (M210465).

Pfizer/BioNTech (BNT162b2) Vaccinees: samples from donors vaccinated with BNT162b2 were used for the pseudovirus neutralization assay against D614G (*N* = 6), Beta (*N* = 6), Delta (*N* = 6), and C.1.2 (*N* = 7) and ADCC activity (*N* = 11) against D614G and C.1.2. These samples were obtained from healthy donors 2 months after their second dose (Supplementary Table 4). Ethics approval for the use of these samples were obtained from the Human Research Ethics Committee of the Faculty of Health Sciences, University of the Witwatersrand (M210465).

Live virus neutralization assay samples: nasopharyngeal and oropharyngeal swab samples and plasma samples were obtained from hospitalized adults with PCR-confirmed SARS-CoV-2 infection who were enrolled in a prospective cohort study approved by the Biomedical Research Ethics Committee at the University of KwaZulu–Natal (reference BREC/00001275/2020). Sixteen of these samples were used in the live virus neutralization assay against Beta and C.1.2 for those infected during the second wave (*N* = 7 Wave 2), the majority (*N* = 6) of whom were confirmed to be infected with the Beta variant; and against C.1.2 and Delta for those infected with Delta during the third wave (*N* = 9 Wave 3) (Supplementary Table 5). For the Pfizer/BioNTech BNT162b2 live virus assay six samples (*N* = 2 females and *N* = 4 males) were collected and provided by the United World Antivirus Research Network (UWARN), participants were between the ages of 20–59 and samples were collected from 2.9 to 5.1 months post second vaccination dose (Supplementary Fig. 6). Ethics approval for the use of these samples was obtained by the Biomedical Research Ethics Committee at the University of KwaZulu–Natal (BREC/00001275/2020).

### SARS-CoV-2 whole-genome sequencing and genome assembly

#### RNA extraction

RNA was extracted either manually or automatically in batches, using the QIAamp viral RNA mini kit (QIAGEN, California, USA) as per manufacturer’s instructions, using the Nucleo Mag Pathogen kit (Macherey-Nagel, Duren, Germany) on a Hamilton Microlab STAR instrument (Hamilton Company, Reno, NV), or on the Chemagic 360 using the CMG-1049 kit (PerkinElmer, Massachusetts, USA). A modification was done on the manual extractions by adding 280 µl per sample, in order to increase yields. Three hundred microliters of each sample was used for automated magnetic bead-based extraction using the Chemagic 360. RNA was eluted in 60 µl of the elution buffer. Isolated RNA was stored at −80 °C prior to use.

#### PCR and library preparation

Sequencing was performed using the Illumina COVIDSeq protocol (Illumina Inc., San Diego, CA, USA) or nCoV-2019 ARTIC network sequencing protocol v3 (https://artic.network/ncov-2019). These are amplicon-based next-generation sequencing approaches. Briefly, for the nCoV-2019 ARTIC network sequencing protocol, the first strand synthesis was carried out on extracted RNA samples using random hexamers primers from the SuperScript IV reverse transcriptase synthesis kit (Life Technologies) or LunaScript RT SuperMix Kit (New England Biolabs (NEB), Ipswich, MA, USA). The synthesized cDNA was amplified using multiplex polymerase chain reactions (PCRs) using ARTIC nCoV-2019 v3 primers. For COVIDSeq protocol, the first strand synthesis was carried using random hexamers primers from Illumina and the synthesized cDNA underwent two separate multiplex PCR reactions.

For Illumina sequencing using the nCoV-2019 ARTIC network sequencing protocol, the pooled PCR products underwent bead-based tagmentation using the Nextera Flex DNA library preparation kit. The adapter-tagged amplicons were cleaned-up using AmpureXP purification beads (Beckman Coulter, High Wycombe, UK) and amplified using one round of PCR. The PCRs were indexed using the Nextera CD indexes (Illumina, Inc.) according to the manufacturer’s instructions. For the COVIDSeq sequencing protocol, pooled PCR-amplified products were processed for tagmentation and adapter ligation using IDT for Illumina Nextera UD Indexes. Further enrichment and cleanup was performed as per protocols provided by the manufacturer (Illumina Inc.). Pooled samples from the both COVIDSeq protocol and the nCoV-2019 ARTIC network protocol were quantified using a Qubit 3.0 or 4.0 fluorometer (Invitrogen Inc., Waltham, MA, USA), using the Qubit dsDNA High Sensitivity assay according to manufacturer’s instructions. The fragment sizes were analyzed using a TapeStation 4200 (Invitrogen Inc.). The pooled libraries were further normalized to 4 nM concentration and 25 μl of each normalized pool containing unique index adapter sets were combined in a new tube. The final library pool was denatured and neutralized with 0.2 N sodium hydroxide and 200 mM Tris-HCL (pH7), respectively. 1.5 pM sample library was spiked with 2% PhiX. Libraries were loaded onto a 300-cycle NextSeq 500/550 HighOutput Kit v2 and run on the Illumina NextSeq 550 instrument (Illumina Inc.).

For Oxford Nanopore sequencing: PCR products were quantified, without prior cleaning, using the Qubit dsDNA High Sensitivity assay on the Qubit 2.0 fluorometer (Thermo Fisher Scientific, Waltham, MA, USA). Following DNA repair (NEB) and end-prep reactions (NEB), up to 96 samples were barcoded by ligation using the EXP-NBD196 kit (Oxford Nanopore Technologies (ONT), Oxford, U.K.). Barcoded samples were pooled, bead-purified and ligated to sequencing adapters using the Adapter Mix II Expansion kit (ONT). After the bead-purification, the DNA concentration was quantified on the Qubit 2.0 instrument (Thermo Fisher). Up to 100 ng of the library was diluted in 75 μl of sequencing mix loaded on an R9 flow-cell (ONT). A sequencing experiment was performed using MinKNOW software on the GridION X5 (ONT), with the high-accuracy base-calling setting. The NC045512 reference (https://www.ncbi.nlm.nih.gov/nuccore/1798174254) was used for alignment during base-calling and the barcodes were split into different folders.

#### Assembly, processing and quality control of genomic sequences

Raw reads from Illumina sequencing were assembled using the Exatype NGS SARS-CoV-2 pipeline v1.6.1, (https://sars-cov-2.exatype.com/) or Genome Detective 1.132/1.133 (https://www.genomedetective.com/) and the Coronavirus Typing Tool^25,26^. Samples sequenced from Oxford Nanopore GridION were assembled according to the ARTIC nCoV-2019 novel coronavirus bioinformatics protocol or using the Fastq QC + ARTIC + NextClade pipeline on Epi2Me (Oxford Nanopore Technologies). For these samples, raw reads were base called and demultiplexed using Guppy. To guarantee accuracy of the base calls, we only used dual indexed reads (i.e., required barcodes at both ends). A reference-based assembly and mapping was generated for each sample using Minimap2 and consensus calculated using Nanopolish. The reference genome used throughout the assembly process was NC_045512.2 (Accession number: MN908947.3, https://www.ncbi.nlm.nih.gov/nuccore/MN908947.3). The initial assembly obtained was cleaned by aligning mapped reads to the references and filtering out low-quality mutations using the Geneious software v2021.0.3 (Biomatters). Quality control reports were obtained from Nextclade^27^. The resulting consensus sequence was further manually polished by considering and correcting indels in homopolymer regions that break the open reading frame (probably sequencing errors) using Aliview v1.27, (http://ormbunkar.se/aliview/)^28^. Mutations resulting in mid-gene stop codons and frameshifts were reverted to wild type. Regions with clustered mutations and deletions resulting in frameshifts were annotated as gaps and insertions were removed. Sequences with less than 80% coverage relative to the Wuhan-Hu-1 reference were discarded. All assemblies were deposited in GISAID (https://www.gisaid.org/)^9^ and the GISAID accession was included as part of Supplementary Table 1. Clade and lineage assignment was determined using Nextclade^27^ and Pangolin^29^.

#### Classification of lineage, clade and associated mutations

The “Phylogenetic Assignment of Named Global Outbreak Lineages” (PANGOLIN) software suite (https://github.com/hCoV-2019/pangolin) was used for the dynamic SARS-CoV-2 lineage classification^29^. The SARS-CoV-2 genomes in our dataset were also classified using the clade classification proposed by Nextstrain (https://nextstrain.org/) built for real-time tracking of the pathogen evolution^30^. The PANGO lineage identified predominantly in South Africa in this study is now assigned to the lineage C.1.2 (Pangolin version v3.1.7, lineages version 2021-07-28); the corresponding Nextclade classification is 20D (Nextclade version v1.5.3, clades version 2021-07-28). The C.1.2 lineage and its associated mutations were further confirmed using the Stanford Coronavirus Antiviral & Resistance Database (CoVDB) (https://covdb.stanford.edu/) and Outbreak.info (https://outbreak.info/).

#### Dataset compilation

At the time of writing, there were over 3.7 million SARS-CoV-2 genomes available on GISAID (https://www.gisaid.org). Due to the size of this dataset, sub-sampling was performed to obtain a representative but manageable sample of genomes. A preliminary dataset was downloaded from GISAID. For C.1.2, genomes were manually curated to include all genomes with more than 90% coverage and complete date information. Due to the dynamic nature of GISAID submissions we did not include sequences that were released later than September 10th, with the exception of the Eswatini sequences (Supplementary Table 2), which we had sequenced and deposited on GISAID (with permission of the originating laboratory) and so could access before their release date. Following this, C.1 (the original lineage to which C.1.2 was assigned), C.1.1 (a Mozambican lineage that evolved from C.1^7^) and South African genomes were downloaded with the options “complete”, “low coverage excluded”, and “collection date complete” selected to ensure that only genomes with complete date information and more than 95% coverage were included. The global and African Auspice datasets were also downloaded (accessed 10 September 2021). This dataset was further down-sampled using a custom build of the Nextstrain SARS-CoV-2 pipeline^30^ to produce a final dataset of 6192 genomes. Of these, 156 are from lineage C.1.2. Due to the fact that C.1.2 was first detected and is most prevalent in South Africa, we chose to include a large proportion of South African sequences, resulting in 1991 South African genomes. To include global context, there were an additional 1047 sequences from the rest of Africa, 902 from Asia, 1132 from Europe, 484 from South America, 430 from North America, and 206 from Oceania. This dataset included genomes from all variants of concern (VOC) and variants of interest (VOI) as defined by the WHO at the time of writing (www.who.int, accessed 17 September 2021).

#### Temporal analysis

Using a dataset of global C.1.2 sequences publicly available on GISAID, we constructed a maximum-likelihood tree in IQ-tree^31^. We inspected this maximum-likelihood tree in TempEst v.1.5.3 for the presence of a temporal (that is, molecular clock) signal^32^. The regression of root-to-tip distance against sampling date showed that the sequences evolved in a relatively strong clock-like manner, with a correlation coefficient of 0.43 and *R*^2^ of 0.18.

#### Phylogenetic analysis and recombination detection

Phylogenetic analysis was conducted with a custom Nextstrain SARS-CoV-2 build (using NextStrain v7)^30^. Briefly, the pipeline filters sequences, aligns these sequences with Nextalign (https://github.com/neherlab/nextalign), sub-samples the datasets (resulting in the dataset described above), constructs a phylogenetic tree with IQ-TREE^31^, refines and dates the tree with TreeTime^33^, reconstructs ancestral states, and assigns Nextstrain clades to the sequences. The tree was visualized with Auspice to confirm the presence of a C.1.2 cluster. This revealed that several non-C.1.2 samples clustered with C.1.2. These sequences were inspected for the presence of the major C.1.2 mutations (dark purple mutations in Supp Fig. 3b). All sequences possessed a subset of the major mutations (a number of sequences had missing data spanning the regions of defining mutations); this, along with the clustering, was used as evidence to re-assign the sequences to C.1.2, resulting in a set of 156 C.1.2 genomes.

To model phylogenetic diffusion, duplicate Markov Chain Monte Carlo (MCMC) analyses were executed in BEAST v1.10.4 for 100 million iterations with sampling every 10,000 steps in the chain. For each sequence, latitude and longitude were attributed to a point randomly sampled within the local area or district of origin. For this analysis, we used the strict molecular clock model, the HKY + I nucleotide substitution model, and the exponential growth coalescent model^34^. Convergence of runs was assessed in Tracer v.1.7.1 based on high effective sample sizes and good mixing^35^. Maximum clade credibility trees for each run were summarized using TreeAnnotator after discarding the first 10% of the chain as burn-in. The R package “seraphim” was used to extract and map the spatiotemporal information embedded in the MCC trees^36^.

We tested for evidence of recombination with RDP5^37^ using seven recombination detection methods implemented therein, and a liberal multiple testing uncorrected *p*-value cutoff of 0.05 to maximize power to detect recombination. Specifically, we screened an alignment of 12 representative C.1.2, eight Alpha sequences, eight Beta sequences, nine Delta sequences and six C.1 sequences for evidence that the mutations shared by the VOCs and C.1.2 had been acquired by recombination between viruses in the VOC and C.1 lineages. The specific sequences selected for analysis were representative of the diversity within their respective lineages in context of the South African epidemic.

#### SARS-CoV-2 model

We modeled the spike protein on the basis of the Protein Data Bank coordinate set 7A94. We used the Pymol program (The PyMOL Molecular Graphics System, version 2.2.0) for visualization.

#### Lentiviral pseudovirus production and neutralization assay

Virus production and pseudovirus neutralization assays were done as previously described^14^. Briefly, 293T/ACE2.MF cells modified to overexpress human ACE2 (kindly provided by M. Farzan (Scripps Research)) were cultured in DMEM (Gibco BRL Life Technologies) containing 10% heat-inactivated serum (FBS) and 3 μg ml^−1^ puromycin at 37 °C, 5% CO_2_. Cell monolayers were disrupted at confluency by treatment with 0.25% trypsin in 1 mM EDTA (Gibco BRL Life Technologies). The SARS-CoV-2 Wuhan-1 spike, cloned into pCDNA3.1, was mutated using the QuikChange Lightning Site-Directed Mutagenesis kit (Agilent Technologies) and NEBuilder HiFi DNA Assembly Master Mix (NEB) to include D614G (wild-type) or lineage defining mutations for Beta (L18F, D80A, D215G, 241-243del, K417N, E484K, N501Y, D614G and A701V), Delta (T19R, 156-157del, R158G, L452R, T478K, D614G, P681R and D950N) and C.1.2 (P9L, C136F, Y144del, R190S, D215G, 242-243del, Y449H, E484K, N501Y, D614G, H655Y, N679K, T716I and T859N). Pseudoviruses were produced by co-transfection in 293T/17 cells with a lentiviral backbone (HIV-1 pNL4.luc encoding the firefly luciferase gene) and either of the SARS-CoV-2 spike plasmids with PEIMAX (Polysciences). Culture supernatants were clarified of cells by a 0.45-μM filter and stored at −70 °C. Plasma samples were heat-inactivated and clarified by centrifugation. Pseudovirus and serially diluted plasma/sera were incubated for 1 h at 37 °C, 5% CO_2_. Cells were added at 1 × 10^4^ cells per well after 72 h of incubation at 37 °C, 5% CO_2_, luminescence was measured using PerkinElmer Life Sciences Model Victor X luminometer. Neutralization was measured as described by a reduction in luciferase gene expression after single-round infection of 293T/ACE2.MF cells with spike-pseudotyped viruses. Titers were calculated as the reciprocal plasma dilution (ID_50_) causing 50% reduction of relative light units.

#### Viral expansion and live virus neutralization assay

All work with live virus was performed in Biosafety Level 3 containment using protocols for SARS-CoV-2 approved by the Africa Health Research Institute Biosafety Committee. ACE2-expressing H1299-E3 cells were used for the initial isolation (P1 stock) followed by passaging in Vero E6 cells (P2 and P3 stocks, where P3 stock was used in experiments). ACE2-expressing H1299-E3 cells were seeded at 1.5 × 10^5^ cells/ml and incubated for 18–20 h. After one DPBS wash, the sub-confluent cell monolayer was inoculated with 500 μl universal transport medium diluted 1:1 with growth medium filtered through a 0.45 μm filter. Cells were incubated for 1 h. Wells were then filled with 3 ml complete growth medium. After 8 days of infection, cells were trypsinized, centrifuged at 300 × *g* for 3 min and resuspended in 4 ml growth medium. Then 1 ml was added to Vero E6 cells that had been seeded at 2 × 10^5^ cells/ml 18–20 h earlier in a T25 flask (approximately 1:8 donor-to-target cell dilution ratio) for cell-to-cell infection. The coculture of ACE2-expressing H1299-E3 and Vero E6 cells was incubated for 1 h and the flask was then filled with 7 ml of complete growth medium and incubated for 6 days. The viral supernatant (P2 stock) was aliquoted and stored at −80 °C and further passaged in Vero E6 cells to obtain the P3 stock used in experiments as follows: a T25 flask (Corning) was seeded with Vero E6 cells at 2 × 10^5^ cells/ml and incubated for 18–20 h. After one DPBS wash, the sub-confluent cell monolayer was inoculated with 500 μl universal transport medium diluted 1:1 with growth medium and filtered through a 0.45 μm filter. Cells were incubated for 1 h. The flask was then filled with 7 ml of complete growth medium. After infection for 4 days, supernatants of the infected culture were collected, centrifuged at 300 × *g* for 3 min to remove cell debris and filtered using a 0.45 μm filter. Viral supernatant was aliquoted and stored at −80 °C.

Vero E6 cells were plated in a 96-well plate (Corning) at 30,000 cells per well 1 day before infection. Approximately 5 ml sterile water was added between wells to prevent wells at the edge drying more rapidly, which we have observed to cause edge effects resulting in lower number of foci. Plasma was separated from EDTA-anticoagulated blood by centrifugation at 500 × *g* for 10 min and stored at −80 °C. Aliquots of plasma samples were heat-inactivated at 56 °C for 30 min and clarified by centrifugation at 10,000×*g* for 5 min, after which the clear middle layer was used for experiments. Inactivated plasma was stored in single-use aliquots to prevent freeze–thaw cycles. For experiments, plasma was serially diluted two-fold from 1:100 to 1:1600; this is the concentration that was used during the virus–plasma incubation step before addition to cells and during the adsorption step. As a positive control, the GenScript A02051 anti-spike monoclonal antibody was added. Virus stocks were used at approximately 50–100 focus-forming units per microwell and added to diluted plasma; antibody–virus mixtures were incubated for 1 h at 37 °C, 5% CO_2_. Cells were infected with 100 μl of the virus–antibody mixtures for 1 h, to allow adsorption of virus. Subsequently, 100 μl of a 1× RPMI 1640 (Sigma-Aldrich, R6504), 1.5% carboxymethylcellulose (Sigma-Aldrich, C4888) overlay was added to the wells without removing the inoculum. Cells were fixed at 18 h after infection using 4% paraformaldehyde (Sigma-Aldrich) for 20 min. For staining of foci, a rabbit anti-spike monoclonal antibody (BS-R2B12, GenScript A02058) was used at 0.5 μg/ml as the primary detection antibody. Antibody was resuspended in a permeabilization buffer containing 0.1% saponin (Sigma-Aldrich), 0.1% BSA (Sigma-Aldrich) and 0.05% Tween-20 (Sigma-Aldrich) in PBS. Plates were incubated with primary antibody overnight at 4 °C, then washed with wash buffer containing 0.05% Tween-20 in PBS. Secondary goat anti-rabbit horseradish peroxidase (Abcam ab205718) antibody was added at 1 μg/ml and incubated for 2 h at room temperature with shaking. The TrueBlue peroxidase substrate (SeraCare 5510-0030) was then added at 50 μl per well and incubated for 20 min at room temperature. Plates were then dried for 2 h and imaged using a Metamorph-controlled Nikon TiE motorized microscope with a 2× objective or ELISPOT instrument with built-in image analysis (C.T.L). For microscopy images, automated image analysis was performed using a custom script in MATLAB v.2019b (Mathworks), in which focus detection was automated and did not involve user curation. Plasma dilutions used were 1:10, 1:20, 1:40, 1:80, 1:160, 1:320, 1:640, and 1:1280 for self-plasma and 1:25, 1:50, 1:100, 1:200, 1:400, 1:800, and 1:1600 for all other plasma samples tested.

#### Antibody-dependent cellular cytotoxicity (ADCC) assay

The ability of plasma antibodies to cross-link FcγRIIIa (CD16) and spike expressing cells was measured as a proxy for ADCC. HEK293T cells were transfected with 5 μg of SARS-CoV-2 wild-type variant spike (D614G), Beta, Delta or C.1.2 spike plasmids using PEI-MAX 40,000 (Polysciences) and incubated for 2 days at 37 °C. Expression of spike was confirmed by binding of CR3022 and P2B-2F6 and their detection by anti-IgG APC (Biolegend, San Diego, California) staining measured by flow cytometry. Subsequently, 1 × 10^5^ spike transfected cells per well were incubated with heat inactivated plasma (1:100 final dilution) or control mAbs (final concentration of 100 μg/ml) in RPMI 1640 media supplemented with 10% FBS 1% Pen/Strep (Gibco, Gaithersburg, MD) for 1 h at 37 °C. Jurkat-Lucia™ NFAT-CD16 cells (Invivogen) (2 × 10^5^ cells/well) were added and incubated for 24 h at 37 °C, 5% CO2. Twenty microliter of supernatant was then transferred to a white 96-well plate with 50 μl of reconstituted QUANTI-Luc secreted luciferase and read immediately on a Victor 3 luminometer with 1 s integration time. Cells were gated on singlets, live cells (determined by Live/dead™ Viability dye; Thermofisher Scientific, Oslo, Norway), and those cells that were positive for IgG and spike specific monoclonal antibodies binding to their surface. Relative light units (RLU) of a no antibody control were subtracted as background. Palivizumab was used as a negative control, while CR3022 was used as a positive control, and P2B-2F6 to differentiate the Beta from the D614G variant. To induce the transgene 1× cell stimulation cocktail (Thermofisher Scientific, Oslo, Norway) and 2 μg/ml ionomycin in R10 was added as a positive control.

#### Statistics and reproducibility

A Wilcoxon matched pairs signed-rank test was used to measure the differences in neutralization ID_50_ titer between matched samples against the different viruses for both the live virus and pseudovirus neutralization assays, as well as the difference in ADCC RLU. *p*-values are reflected in the graphs, represented as an asterix where: * represents *p* < 0.05, ** represents *p* < 0.01, *** represents *p* < 0.001 and **** represents *p* < 0.0001.

### Reporting summary

Further information on research design is available in the Nature Research Reporting Summary linked to this article.

## Supplementary information

Supplementary Information

Peer Review File

Description of Additional Supplementary Files

Supplementary Software 1

Reporting Summary

## Data Availability

All of the global SARS-CoV-2 genomes generated and presented in this article are publicly accessible through the GISAID^9^ platform (https://www.gisaid.org/), along with all other SARS-CoV-2 genomes generated by the NGS-SA. The GISAID accession identifiers of the C.1.2 sequences analyzed in this study are provided as part of Supplementary Tables 1 and 2, which also contain the metadata for the sequences. The Nextstrain build of C.1.2 and global sequences is available at https://nextstrain.org/groups/ngs-sa/COVID19-C.1.2-2022-01-05. The GISAID accession identifiers for the full set of sequences used in this build can be accessed at https://github.com/NICD-CRDM/C.1.2_scripts/tree/main/Nextstrain_files. The GISAID accession identifiers for the sequences used in Supp. Fig. 2a and temporal analysis can be accessed at https://github.com/NICD-CRDM/C.1.2_scripts in the files violin_plot_IDs.xlsx and C.1.2_global_tempest.xlsx respectively. The shapefile used for South African maps in Supplementary Fig. 1 was downloaded from https://gadm.org/ (licensed for use in academic publications, see https://gadm.org/license.html) and visualised in R with ggplot2. The global map in Supplementary Fig. 1 was obtained from the rnaturalearth package (public domain, see https://docs.ropensci.org/rnaturalearth/articles/rnaturalearth.html) and visualised with ggplot2. The data was based on sequences available on GISAID at the time.

## Code availability

The R and python scripts used to generate figures (excluding bar charts) in this paper are available at https://github.com/NICD-CRDM/C.1.2_scripts. The Nextstrain build profile, other scripts required to run the custom pipeline, and GISAID accession identifiers for all sequences in the final tree are available at https://github.com/NICD-CRDM/C.1.2_scripts/tree/main/Nextstrain_files. The MATLAB scripts used for microscopy are available at https://github.com/NICD-CRDM/C.1.2_scripts/tree/main/microscopy. This code is also available in Supplementary Software 1.
