# Supplementary material for: Emergence and phenotypic characterization of the global SARS-CoV-2 C.1.2 lineage
Source: Nat Commun. 2022 Apr 8;13:1976. doi: 10.1038/s41467-022-29579-9 (PMC8993834; doi:10.1038/s41467-022-29579-9)
Supplement: Supplementary file 1 — Supplementary Information [file 41467_2022_29579_MOESM1_ESM.pdf]

SUPPLEMENTAL INFORMATION

Emergence and phenotypic characterization of the global SARS-CoV-2 C.1.2 lineage

Author list

Cathrine Scheepers[#1,2], Josie Everatt[#1], Daniel G. Amoako[1], Houriiyah Tegally[3], Constantinos Kurt Wibmer[1], Anele Mnguni[1], Arshad Ismail[1], Boitshoko Mahlangu[1], Bronwen E. Lambson[1,2], Darren P. Martin[4], Eduan Wilkinson,[3,5], Emmanuel James San[3], Jennifer Giandhari[3], Nelia Manamela[1,2], Noxolo Ntuli[1], Prudence Kgagudi[1,2], Sandile Cele[6,7], Simone I. Richardson[1,2], Sureshnee Pillay[3], Thabo Mohale[1], Upasana Ramphal[3], Yeshnee Naidoo[3], Zamantungwa T. Khumalo[1,8], Gaurav Kwatra[9,10], Glenda Gray[11], Linda-Gail Bekker[12], Shabir A. Madhi[9], Vicky Baillie[9], Wesley C. Van Voorhis[13], Florette K. Treurnicht[14,15], Marietjie Venter[16], Koleka Mlisana[14], Nicole Wolter[1,15], Alex Sigal[7,7,17], Carolyn Williamson[4,14 18], Nei-yuan Hsiao[4,14,18], Nokukhanya Msomi[14,19], Tongai Maponga[20], Wolfgang Preiser[20,14], Zinhle Makatini[14,15], Richard Lessells[3,5], Penny L. Moore[1,2,4,21], Tulio de Oliveira[3,5], Anne von Gottberg[1,15,22] and Jinal N. Bhiman*[1,15]

Affiliations

[1]National Institute for Communicable Diseases (NICD) of the National Health Laboratory Service (NHLS), 2131, Johannesburg, South Africa. [2]SA MRC Antibody Immunity Research Unit, School of Pathology, Faculty of Health Sciences, University of the Witwatersrand, 2132, Johannesburg, South Africa. [3]KwaZulu-Natal Research Innovation and Sequencing Platform (KRISP), Nelson R Mandela School of Medicine, University of KwaZulu-Natal, 4001, Durban, South Africa. [4]Institute of Infectious Disease and Molecular Medicine, University of Cape Town, 7925, Cape Town, South Africa. [5]Centre for Epidemic Response and Innovation (CERI), School of Data Science and Computational Thinking, Stellenbosch University, 7600, Stellenbosch, South Africa. [6]Africa Health Research Institute, 4001, Durban, South Africa. [7]School of Laboratory Medicine and Medical Sciences, University of KwaZulu-Natal, 4041, Durban, South Africa. [8]Department of Veterinary Tropical Diseases, Faculty of Veterinary Science, University of Pretoria, 0110, Onderstepoort, South Africa. [9]South African Medical Research Council Vaccines and Infectious Diseases Analytics Research Unit, University of the Witwatersrand, 2000, Johannesburg, South Africa. [10]Department of Clinical Microbiology, Christian Medical College, 632004, Vellore, India. [11]South African Medical Research Council, 7505, Cape Town, South Africa. [12]Desmond Tutu HIV Centre, 7925, Cape Town, South Africa. [13]Center for Emerging and Re-emerging Infectious Diseases (CERID), University of Washington, 98195, Washington, United States. [14]National Health Laboratory Service (NHLS), 2131, Johannesburg, South Africa. [15]Faculty of Health Sciences, University of the Witwatersrand, 2193, Johannesburg, South Africa. [16]Zoonotic Arbo and Respiratory virus research programme, Department Medical Virology, 0002, University of Pretoria. [17]Max Planck Institute for Infection Biology, 10117, Berlin, Germany. [18]Wellcome Centre for Infectious Diseases Research in Africa (CIDRI-Africa), 7925, Cape Town, South Africa. [19]Discipline of Virology, University of KwaZulu-Natal, 4041, Durban, South Africa. [20]Division of Medical Virology, Faculty of Medicine and Health Sciences, Stellenbosch University, Tygerberg, 7602, Cape Town, South Africa. [21]Centre for the AIDS Programme of Research in South Africa (CAPRISA), 4013, KwaZulu-Natal, South Africa. [22]Faculty of Health Sciences, University of Cape Town, 7935, Cape Town, South Africa. .

[#] These authors contributed equally

*Corresponding Author: Jinal N. Bhiman (1 Modderfontein Road, Sandringham, 2131, Gauteng, South Africa; jinalb@nicd.ac.za)

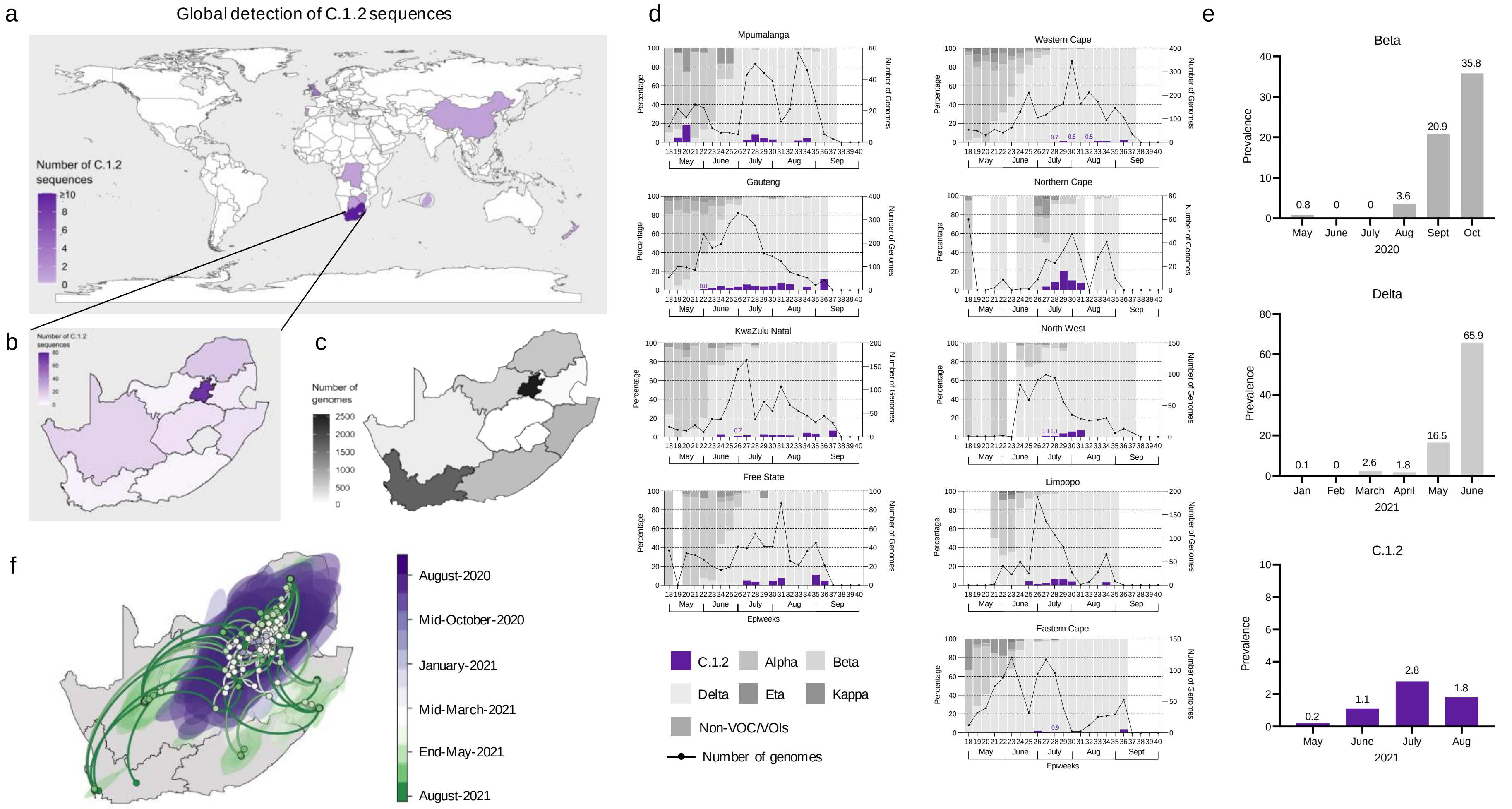

Supplementary Figure 1: Distribution and prevalence of C.1.2 globally. a, Global map highlighting South Africa, Botswana, the Democratic Republic of the Congo, China, England, Eswatini, Mauritius (shown in the magnified bubble), New Zealand, Portugal, Switzerland and Zimbabwe, across which 166 C.1.2 sequences have been detected. Countries coloured in shades of purple according to the number of sequences detected, as shown by the key. b, Map of South Africa highlighting the provinces in which C.1.2 has been detected, colored in shades of purple according to the number of sequences detected, as shown by the key. c, Map of South Africa showing the number of SARS-CoV-2 genomes (N=8,337 as of September 10, 2021) that have been sequenced by province in

the months of May to August 2021. Provinces are coloured in shades of grey according as shown by the key. d, Percentage of genomes that are assigned to various SARS-CoV-2 lineages (coloured in greyscale as shown in the key, where Non-VOC (Variant Of Concern)/VOI (Variant Of Interest) represent ancestral variants by epidemiological week (epiweek) in South Africa for each of the provinces, with C.1.2 shown in purple (based on data submitted to GISAID on September, 21, 2021). The number of genomes sequenced for each epiweek is shown by the black line. e, Early prevalence rates of Beta, Delta and C.1.2 in South Africa based on the number of SARS-CoV-2 sequences generated for each month and submitted to GISAID on September 21, 2021. Bars are coloured by variant as shown in the key for panel d. f, Spatiotemporal reconstruction of the spread of the C.1.2 cluster in South Africa during the third epidemic wave. Circles represent nodes of the maximum clade credibility phylogeny and are colored according to their inferred time of occurrence as shown in the key. Shaded areas represent the 80% highest posterior density interval and depict the uncertainty of the phylogeographic estimates for each node. Solid curved lines denote the links between nodes and the directionality of movement in an anticlockwise direction along the curve.

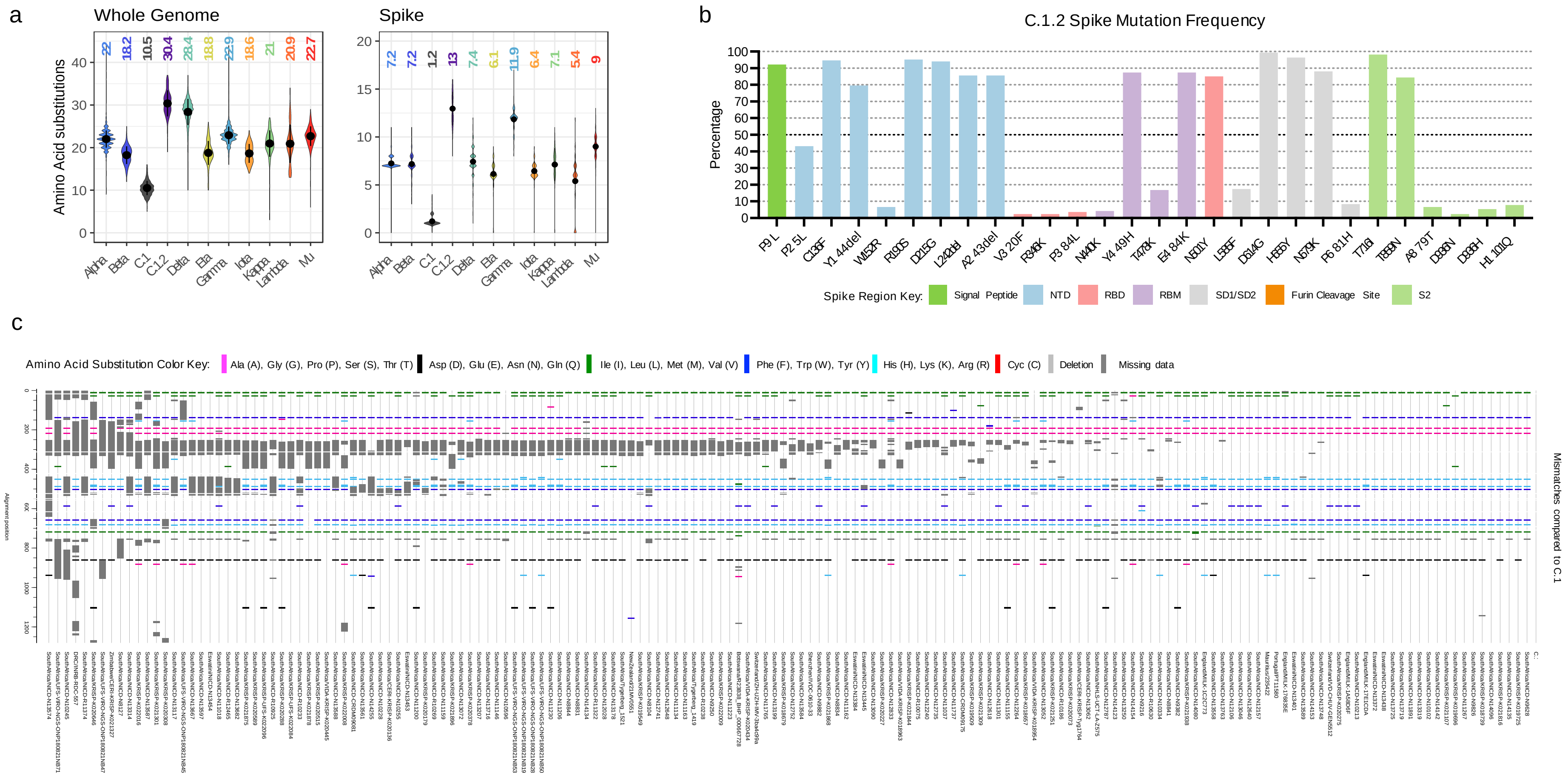

Supplementary Figure 2: C.1.2 mutation profile. a, Whole genome (left panel) and spike region only (right panel) amino acid substitutions across different VOIs and VOCs compared to C.1 (the precursor of C.1.2) and C.1.2, coloured according to nextstrain clade, with C.1.2 in purple and C.1 in blue. The dot and error plots for each variant represent the mean and range of amino acid changes for each variant. Samples sizes for each variant varied with Alpha=3685, Beta=344, C.1=271, C.1.2=102, Delta=2603, Eta=189, Gamma=866, Iota=315, Kappa=210, Lambda=154 and Mu=585. b, Frequency of spike mutations across C.1.2 sequences, though frequencies of these mutations may be underrepresented due to high levels of missing data (represented by dark grey blocks shown in panel c). Regions within the spike are colored according to region as shown in the key including the N-terminal domain (NTD, blue), receptor binding domain

(RBD, red), receptor binding motif (RBM, purple) and subdomain 1 and 2 (SD1 or SD2, grey). c, Highlighter plot of C.1.2 spike sequences with (N=166) identified across the globe, labelled according to the location identified and sequence name. Mismatches compared to the C.1 strain (top/master sequence) are colored by Se-Al in the hiv.lanl.gov highlighter tool as shown in the key. Large dark grey regions represent missing sequence data. Spike regions are also coloured according to the key in panel b.

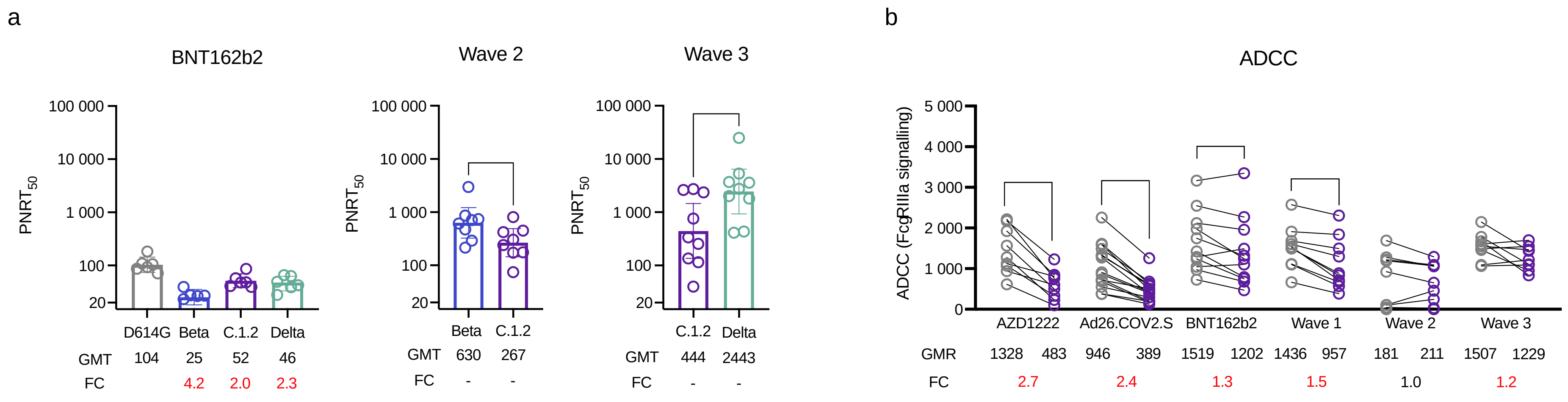

Supplementary Figure 3: Live virus neutralization and ADCC profiles of C.1.2. a, Neutralization activity measure by the live virus neutralization assay from biologically independent donor samples either vaccinated with BNT162b2 (N=6) or previously infected during the second (N=8) and third waves (N=9) in South Africa against the wild-type (D614G), Beta, Delta and C.1.2 variants. Bar graphs represent the geometric mean titer (GMT) for each group with the error bars representing the 95% confidence intervals, dots represent individual sample titers. Statistical significance based on the Wilcoxon two-tailed matched-pairs signed rank test are shown above graphs. The ** denotes p-values p<0.01. No adjustments for multiple testing were made. GMT and fold-change (FC) differences relative to D614G (wild-type) are given below the graph, with red representing decrease in titre and green representing increases in titre. b, Antibody Dependent Cellular Cytotoxicity (ADCC) activity represented as relative light units (RLU) from biologically independent plasma samples from donors either vaccinated with AZD1222 (N=9), Ad26.COV.2.S (N=15) or BNT162b2 (N=11) or previously infected during waves one (N=11), two (N=10) or three (N=9) in South Africa against the wild-type (D614G, shown as grey circles) and C.1.2 (shown as purple circles). Statistical significance based on the Wilcoxon two-tailed matched-pairs signed rank test are shown above graphs. P-values are denoted with "*" symbols: * p<0.05 and **** p<0.0001. Geometric mean of the RLU (GMR) and fold-changes (FC) relative to the wild-type (D614G) are given below the graph with red representing decreases in RLU and black representing no difference.

Supplementary Table 1: Reference set of C.1.2 genomes on GISAID from South Africa. Provided are the GISAID strain name and GISAID_EPI_ISL accession numbers for all C.1.2 sequences detected in South Africa as of data deposited on September 10, 2021. Provided are the provincial breakdown, vaccination breakthrough status and use in various analyses.

Virus Name	GISAID_EPI_ISL Number	Province	Vaccine Breakthrough	Use in analysis
hCoV-19/SouthAfrica/CERI-KRISP-K020136/2021	EPI_ISL_3267751	Eastern Cape	Yes	Tree, temporal analysis, highlighter plot
hCoV-19/SouthAfrica/CERI-KRISP-Tyg1764/2021	EPI_ISL_3827640	Western Cape Province	Unknown	Tree, temporal analysis, highlighter plot
hCoV-19/SouthAfrica/KRISP-K018657/2021	EPI_ISL_2726854	Gauteng	Unknown	Tree, temporal analysis, highlighter plot
hCoV-19/SouthAfrica/KRISP-K018679/2021	EPI_ISL_2726855	Gauteng	Unknown	Tree, temporal analysis, highlighter plot
hCoV-19/SouthAfrica/KRISP-K018739/2021	EPI_ISL_2770450	Gauteng	Unknown	Tree, temporal analysis, highlighter plot
hCoV-19/SouthAfrica/KRISP-K019509/2021	EPI_ISL_3132529	Gauteng	Unknown	Tree, temporal analysis, highlighter plot
hCoV-19/SouthAfrica/KRISP-K019549/2021	EPI_ISL_3132566	Gauteng	Unknown	Tree, temporal analysis, highlighter plot
hCoV-19/SouthAfrica/KRISP-K019696/2021	EPI_ISL_3132608	KwaZulu-Natal	Unknown	Tree, temporal analysis, highlighter plot
hCoV-19/SouthAfrica/KRISP-K019725/2021	EPI_ISL_3132623	Gauteng	Unknown	Tree, temporal analysis, highlighter plot
hCoV-19/SouthAfrica/KRISP-K020073/2021	EPI_ISL_3729063	Gauteng	Unknown	Tree, temporal analysis, highlighter plot
hCoV-19/SouthAfrica/KRISP-K020179/2021	EPI_ISL_3267757	KwaZulu-Natal	Unknown	Tree, temporal analysis, highlighter plot
hCoV-19/SouthAfrica/KRISP-K020227/2021	EPI_ISL_3447713	Gauteng	Unknown	Tree, temporal analysis, highlighter plot
hCoV-19/SouthAfrica/KRISP-K020275/2021	EPI_ISL_3447714	Gauteng	Unknown	Tree, temporal analysis, highlighter plot
hCoV-19/SouthAfrica/KRISP-K020288/2021	EPI_ISL_3447773	Gauteng	Unknown	Tree, temporal analysis, highlighter plot
hCoV-19/SouthAfrica/KRISP-K020308/2021	EPI_ISL_3261918	Gauteng	Unknown	Tree, temporal analysis, highlighter plot
hCoV-19/SouthAfrica/KRISP-K020378/2021	EPI_ISL_3261970	KwaZulu-Natal	Unknown	Tree, temporal analysis, highlighter plot
hCoV-19/SouthAfrica/KRISP-K020515/2021	EPI_ISL_3730315	KwaZulu-Natal	Unknown	Tree, temporal analysis, highlighter plot
hCoV-19/SouthAfrica/KRISP-K020589/2021	EPI_ISL_3447758	KwaZulu-Natal	Unknown	Tree, temporal analysis, highlighter plot
hCoV-19/SouthAfrica/KRISP-K020646/2021	EPI_ISL_3447717	KwaZulu-Natal	Unknown	Highlighter plot
hCoV-19/SouthAfrica/KRISP-K021107/2021	EPI_ISL_3663539	KwaZulu-Natal	Unknown	Tree, temporal analysis, highlighter plot
hCoV-19/SouthAfrica/KRISP-K021251/2021	EPI_ISL_3722231	Gauteng	Unknown	Tree, temporal analysis, highlighter plot
hCoV-19/SouthAfrica/KRISP-K021301/2021	EPI_ISL_3722264	Gauteng	Unknown	Tree, temporal analysis, highlighter plot
hCoV-19/SouthAfrica/KRISP-K021309/2021	EPI_ISL_3722270	Gauteng	Unknown	Tree, temporal analysis, highlighter plot
hCoV-19/SouthAfrica/KRISP-K021816/2021	EPI_ISL_3799163	KwaZulu-Natal	Unknown	Tree, temporal analysis, highlighter plot
hCoV-19/SouthAfrica/KRISP-K021844/2021	EPI_ISL_3799102	KwaZulu-Natal	Unknown	Tree, temporal analysis, highlighter plot
hCoV-19/SouthAfrica/KRISP-K021868/2021	EPI_ISL_3799095	Gauteng	Unknown	Tree, temporal analysis, highlighter plot
hCoV-19/SouthAfrica/KRISP-K021875/2021	EPI_ISL_3799137	Gauteng	Unknown	Tree, temporal analysis, highlighter plot
hCoV-19/SouthAfrica/KRISP-K021878/2021	EPI_ISL_3799035	Gauteng	Unknown	Tree, temporal analysis, highlighter plot
hCoV-19/SouthAfrica/KRISP-K021896/2021	EPI_ISL_3799162	Gauteng	Unknown	Tree, temporal analysis, highlighter plot
hCoV-19/SouthAfrica/KRISP-K021938/2021	EPI_ISL_3859884	Gauteng	Unknown	Tree, temporal analysis, highlighter plot
hCoV-19/SouthAfrica/KRISP-K022008/2021	EPI_ISL_3860060	Gauteng	Unknown	Tree, temporal analysis, highlighter plot

hCoV-19/SouthAfrica/KRISP-K022009/2021	EPI_ISL_3860015	Gauteng	Unknown	Tree, temporal analysis, highlighter plot
hCoV-19/SouthAfrica/KRISP-K022016/2021	EPI_ISL_3859890	Gauteng	Unknown	Tree, temporal analysis, highlighter plot
hCoV-19/SouthAfrica/KRISP-UFS-K022084/2021	EPI_ISL_4003563	Free State	Unknown	Tree, temporal analysis, highlighter plot
hCoV-19/SouthAfrica/KRISP-UFS-K022096/2021	EPI_ISL_4003556	Free State	Unknown	Tree, temporal analysis, highlighter plot
hCoV-19/SouthAfrica/NHLS-UCT-LA-Z575/2021	EPI_ISL_3506424	Western Cape	Unknown	Tree, temporal analysis, highlighter plot
hCoV-19/SouthAfrica/NICD-CRDM09081/2021	EPI_ISL_3281601	Gauteng	Unknown	Tree, temporal analysis, highlighter plot
hCoV-19/SouthAfrica/NICD-CRDM09175/2021	EPI_ISL_3281600	Gauteng	Unknown	Tree, temporal analysis, highlighter plot
hCoV-19/SouthAfrica/NICD-N10102/2021	EPI_ISL_3717994	Gauteng	Unknown	Tree, temporal analysis, highlighter plot
hCoV-19/SouthAfrica/NICD-N10213/2021	EPI_ISL_2984801	Gauteng	Yes	Tree, temporal analysis, highlighter plot
hCoV-19/SouthAfrica/NICD-N10228/2021	EPI_ISL_2988404	Gauteng	Unknown	Tree, temporal analysis, highlighter plot
hCoV-19/SouthAfrica/NICD-N10245/2021	EPI_ISL_2988878	Gauteng	Unknown	Highlighter plot
hCoV-19/SouthAfrica/NICD-N10255/2021	EPI_ISL_2988405	Gauteng	Unknown	Tree, temporal analysis, highlighter plot
hCoV-19/SouthAfrica/NICD-N10334/2021	EPI_ISL_3149307	Gauteng	Unknown	Tree, temporal analysis, highlighter plot
hCoV-19/SouthAfrica/NICD-N10596/2021	EPI_ISL_2988409	Gauteng	Unknown	Tree, temporal analysis, highlighter plot
hCoV-19/SouthAfrica/NICD-N11018/2021	EPI_ISL_3149312	Limpopo	Unknown	Highlighter plot
hCoV-19/SouthAfrica/NICD-N11037/2021	EPI_ISL_3149313	Limpopo	Unknown	Tree, temporal analysis, highlighter plot
hCoV-19/SouthAfrica/NICD-N11134/2021	EPI_ISL_3237084	Gauteng	Unknown	Tree, temporal analysis, highlighter plot
hCoV-19/SouthAfrica/NICD-N11146/2021	EPI_ISL_3237092	Gauteng	Unknown	Tree, temporal analysis, highlighter plot
hCoV-19/SouthAfrica/NICD-N11155/2021	EPI_ISL_3237098	Gauteng	Unknown	Tree, temporal analysis, highlighter plot
hCoV-19/SouthAfrica/NICD-N11159/2021	EPI_ISL_3236951	Gauteng	Unknown	Tree, temporal analysis, highlighter plot
hCoV-19/SouthAfrica/NICD-N11162/2021	EPI_ISL_3237100	Gauteng	Unknown	Tree, temporal analysis, highlighter plot
hCoV-19/SouthAfrica/NICD-N11163/2021	EPI_ISL_3236953	Gauteng	Unknown	Tree, temporal analysis, highlighter plot
hCoV-19/SouthAfrica/NICD-N11200/2021	EPI_ISL_3101505	Gauteng	Unknown	Tree, temporal analysis, highlighter plot
hCoV-19/SouthAfrica/NICD-N11206/2021	EPI_ISL_3149299	Gauteng	Unknown	Tree, temporal analysis, highlighter plot
hCoV-19/SouthAfrica/NICD-N11223/2021	EPI_ISL_3149300	Gauteng	Unknown	Tree, temporal analysis, highlighter plot
hCoV-19/SouthAfrica/NICD-N11230/2021	EPI_ISL_3149301	Gauteng	Unknown	Tree, temporal analysis, highlighter plot
hCoV-19/SouthAfrica/NICD-N11267/2021	EPI_ISL_3074033	Gauteng	Unknown	Tree, temporal analysis, highlighter plot
hCoV-19/SouthAfrica/NICD-N11301/2021	EPI_ISL_3149306	Gauteng	Yes	Tree, temporal analysis, highlighter plot
hCoV-19/SouthAfrica/NICD-N11737/2021	EPI_ISL_3838300	Limpopo	Unknown	Tree, temporal analysis, highlighter plot
hCoV-19/SouthAfrica/NICD-N11765/2021	EPI_ISL_3838321	Northern Cape	Unknown	Tree, temporal analysis, highlighter plot
hCoV-19/SouthAfrica/NICD-N11891/2021	EPI_ISL_3411681	Gauteng	Unknown	Tree, temporal analysis, highlighter plot
hCoV-19/SouthAfrica/NICD-N12028/2021	EPI_ISL_3451144	Limpopo	Unknown	Tree, temporal analysis, highlighter plot
hCoV-19/SouthAfrica/NICD-N12106/2021	EPI_ISL_3838375	Eastern Cape	Unknown	Tree, temporal analysis, highlighter plot
hCoV-19/SouthAfrica/NICD-N12157/2021	EPI_ISL_3237233	Eastern Cape	Unknown	Tree, temporal analysis, highlighter plot
hCoV-19/SouthAfrica/NICD-N12240/2021	EPI_ISL_3411589	Gauteng	Unknown	Tree, temporal analysis, highlighter plot
hCoV-19/SouthAfrica/NICD-N12264/2021	EPI_ISL_3237237	Limpopo	Unknown	Tree, temporal analysis, highlighter plot

hCoV-19/SouthAfrica/NICD-N12618/2021	EPI_ISL_3451195	Gauteng	Unknown	Tree, temporal analysis, highlighter plot
hCoV-19/SouthAfrica/NICD-N12640/2021	EPI_ISL_3451214	Gauteng	Unknown	Tree, temporal analysis, highlighter plot
hCoV-19/SouthAfrica/NICD-N12648/2021	EPI_ISL_3451222	Limpopo	Unknown	Tree, temporal analysis, highlighter plot
hCoV-19/SouthAfrica/NICD-N12735/2021	EPI_ISL_3451295	Northern Cape	Unknown	Tree, temporal analysis, highlighter plot
hCoV-19/SouthAfrica/NICD-N12741/2021	EPI_ISL_3451301	Northern Cape	Unknown	Tree, temporal analysis, highlighter plot
hCoV-19/SouthAfrica/NICD-N12752/2021	EPI_ISL_3411463	Gauteng	Unknown	Tree, temporal analysis, highlighter plot
hCoV-19/SouthAfrica/NICD-N12787/2021	EPI_ISL_3411467	Gauteng	Unknown	Tree, temporal analysis, highlighter plot
hCoV-19/SouthAfrica/NICD-N12833/2021	EPI_ISL_3411457	Limpopo	Unknown	Tree, temporal analysis, highlighter plot
hCoV-19/SouthAfrica/NICD-N13046/2021	EPI_ISL_3451358	Limpopo	Unknown	Tree, temporal analysis, highlighter plot
hCoV-19/SouthAfrica/NICD-N13052/2021	EPI_ISL_3451362	Limpopo	Unknown	Tree, temporal analysis, highlighter plot
hCoV-19/SouthAfrica/NICD-N13062/2021	EPI_ISL_3451369	Limpopo	Unknown	Tree, temporal analysis, highlighter plot
hCoV-19/SouthAfrica/NICD-N13072/2021	EPI_ISL_3451378	Limpopo	Unknown	Tree, temporal analysis, highlighter plot
hCoV-19/SouthAfrica/NICD-N13090/2021	EPI_ISL_3451391	Limpopo	Unknown	Tree, temporal analysis, highlighter plot
hCoV-19/SouthAfrica/NICD-N13117/2021	EPI_ISL_3411459	Limpopo	Unknown	Tree, temporal analysis, highlighter plot
hCoV-19/SouthAfrica/NICD-N13178/2021	EPI_ISL_3643965	Mpumalanga	Unknown	Tree, temporal analysis, highlighter plot
hCoV-19/SouthAfrica/NICD-N13199/2021	EPI_ISL_3643862	Mpumalanga	Unknown	Tree, temporal analysis, highlighter plot
hCoV-19/SouthAfrica/NICD-N13207/2021	EPI_ISL_3643860	Mpumalanga	Unknown	Tree, temporal analysis, highlighter plot
hCoV-19/SouthAfrica/NICD-N13250/2021	EPI_ISL_3411458	Limpopo	Unknown	Tree, temporal analysis, highlighter plot
hCoV-19/SouthAfrica/NICD-N13314/2021	EPI_ISL_3643903	Limpopo	Unknown	Tree, temporal analysis, highlighter plot
hCoV-19/SouthAfrica/NICD-N13319/2021	EPI_ISL_3717995	Limpopo	Unknown	Tree, temporal analysis, highlighter plot
hCoV-19/SouthAfrica/NICD-N13558/2021	EPI_ISL_4030025	Northern Cape	Unknown	Tree, temporal analysis, highlighter plot
hCoV-19/SouthAfrica/NICD-N13561/2021	EPI_ISL_4029946	Northern Cape	Unknown	Tree, temporal analysis, highlighter plot
hCoV-19/SouthAfrica/NICD-N13574/2021	EPI_ISL_4030281	Northern Cape	Unknown	Highlighter plot
hCoV-19/SouthAfrica/NICD-N13584/2021	EPI_ISL_4030022	Northern Cape	Unknown	Tree, temporal analysis, highlighter plot
hCoV-19/SouthAfrica/NICD-N13587/2021	EPI_ISL_4029964	Northern Cape	Unknown	Tree, temporal analysis, highlighter plot
hCoV-19/SouthAfrica/NICD-N13588/2021	EPI_ISL_4029943	Northern Cape	Unknown	Tree, temporal analysis, highlighter plot
hCoV-19/SouthAfrica/NICD-N13589/2021	EPI_ISL_4029948	Northern Cape	Unknown	Tree, temporal analysis, highlighter plot
hCoV-19/SouthAfrica/NICD-N13597/2021	EPI_ISL_4029923	North West	Unknown	Tree, temporal analysis, highlighter plot
hCoV-19/SouthAfrica/NICD-N13667/2021	EPI_ISL_3838489	Limpopo	Unknown	Tree, temporal analysis, highlighter plot
hCoV-19/SouthAfrica/NICD-N13682/2021	EPI_ISL_3643842	North West	Unknown	Tree, temporal analysis, highlighter plot
hCoV-19/SouthAfrica/NICD-N13716/2021	EPI_ISL_3838512	Northern Cape	Unknown	Tree, temporal analysis, highlighter plot
hCoV-19/SouthAfrica/NICD-N13719/2021	EPI_ISL_3838515	Northern Cape	Unknown	Tree, temporal analysis, highlighter plot
hCoV-19/SouthAfrica/NICD-N13725/2021	EPI_ISL_3838520	Northern Cape	Unknown	Tree, temporal analysis, highlighter plot
hCoV-19/SouthAfrica/NICD-N13748/2021	EPI_ISL_3838541	Northern Cape	Unknown	Tree, temporal analysis, highlighter plot
hCoV-19/SouthAfrica/NICD-N13763/2021	EPI_ISL_3838556	Northern Cape	Unknown	Tree, temporal analysis, highlighter plot
hCoV-19/SouthAfrica/NICD-N14080/2021	EPI_ISL_3838621	Gauteng	Yes	Tree, temporal analysis, highlighter plot

hCoV-19/SouthAfrica/NICD-N14096/2021	EPI_ISL_3838634	Gauteng	Yes	Tree, temporal analysis, highlighter plot
hCoV-19/SouthAfrica/NICD-N14123/2021	EPI_ISL_3717982	Gauteng	Yes	Tree, temporal analysis, highlighter plot
hCoV-19/SouthAfrica/NICD-N14134/2021	EPI_ISL_3717911	Gauteng	Yes	Tree, temporal analysis, highlighter plot
hCoV-19/SouthAfrica/NICD-N14135/2021	EPI_ISL_3718000	Gauteng	Unknown	Tree, temporal analysis, highlighter plot
hCoV-19/SouthAfrica/NICD-N14142/2021	EPI_ISL_3717932	Gauteng	Unknown	Tree, temporal analysis, highlighter plot
hCoV-19/SouthAfrica/NICD-N14153/2021	EPI_ISL_3717972	Gauteng	Yes	Tree, temporal analysis, highlighter plot
hCoV-19/SouthAfrica/NICD-N14154/2021	EPI_ISL_3717993	Gauteng	Unknown	Tree, temporal analysis, highlighter plot
hCoV-19/SouthAfrica/NICD-N14255/2021	EPI_ISL_3838569	Free State	Unknown	Tree, temporal analysis, highlighter plot
hCoV-19/SouthAfrica/NICD-N8104/2021	EPI_ISL_2695610	Mpumalanga	Unknown	Tree, temporal analysis, highlighter plot
hCoV-19/SouthAfrica/NICD-N8127/2021	EPI_ISL_2695631	Mpumalanga	Unknown	Highlighter plot
hCoV-19/SouthAfrica/NICD-N8831/2021	EPI_ISL_3342730	Gauteng	Unknown	Tree, temporal analysis, highlighter plot
hCoV-19/SouthAfrica/NICD-N8834/2021	EPI_ISL_3342731	Gauteng	Unknown	Tree, temporal analysis, highlighter plot
hCoV-19/SouthAfrica/NICD-N8841/2021	EPI_ISL_3342732	Gauteng	Unknown	Tree, temporal analysis, highlighter plot
hCoV-19/SouthAfrica/NICD-N8844/2021	EPI_ISL_3342733	Gauteng	Unknown	Tree, temporal analysis, highlighter plot
hCoV-19/SouthAfrica/NICD-N9216/2021	EPI_ISL_3342734	Gauteng	Unknown	Tree, temporal analysis, highlighter plot
hCoV-19/SouthAfrica/NICD-N9250/2021	EPI_ISL_3342735	Gauteng	Unknown	Tree, temporal analysis, highlighter plot
hCoV-19/SouthAfrica/NICD-N9382/2021	EPI_ISL_2828749	Gauteng	Unknown	Tree, temporal analysis, highlighter plot
hCoV-19/SouthAfrica/NICD-N9628/2021	EPI_ISL_2827937	Limpopo	Unknown	Tree, temporal analysis, highlighter plot
hCoV-19/SouthAfrica/NICD-N9826/2021	EPI_ISL_2942287	Gauteng	Unknown	Tree, temporal analysis, highlighter plot
hCoV-19/SouthAfrica/NICD-N9882/2021	EPI_ISL_3643966	Gauteng	Unknown	Tree, temporal analysis, highlighter plot
hCoV-19/SouthAfrica/NICD-R10075/2021	EPI_ISL_4029912	North West	Unknown	Tree, temporal analysis, highlighter plot
hCoV-19/SouthAfrica/NICD-R10186/2021	EPI_ISL_4030023	Gauteng	Unknown	Tree, temporal analysis, highlighter plot
hCoV-19/SouthAfrica/NICD-R10233/2021	EPI_ISL_4029941	Gauteng	Unknown	Tree, temporal analysis, highlighter plot
hCoV-19/SouthAfrica/NICD-R10238/2021	EPI_ISL_4030024	Gauteng	Unknown	Tree, temporal analysis, highlighter plot
hCoV-19/SouthAfrica/NICD-R10269/2021	EPI_ISL_4030021	Mpumalanga	Unknown	Tree, temporal analysis, highlighter plot
hCoV-19/SouthAfrica/NICD-R10630/2021	EPI_ISL_3219805	Gauteng	Unknown	Tree, temporal analysis, highlighter plot
hCoV-19/SouthAfrica/NICD-R10925/2021	EPI_ISL_3219868	Limpopo	Unknown	Tree, temporal analysis, highlighter plot
hCoV-19/SouthAfrica/NICD-R11322/2021	EPI_ISL_3451544	KwaZulu-Natal	Unknown	Tree, temporal analysis, highlighter plot
hCoV-19/SouthAfrica/NICD-R11374/2021	EPI_ISL_3451569	North West	Unknown	Tree, temporal analysis, highlighter plot
hCoV-19/SouthAfrica/NICD-R12465/2021	EPI_ISL_4029935	Cape Town	Unknown	Tree, temporal analysis, highlighter plot
hCoV-19/SouthAfrica/Tygerberg_1419/2021	EPI_ISL_3118719	Western Cape	Unknown	Tree, temporal analysis, highlighter plot
hCoV-19/SouthAfrica/Tygerberg_1521/2021	EPI_ISL_3482519	Western Cape	Unknown	Tree, temporal analysis, highlighter plot
hCoV-19/SouthAfrica/UFS-VIRO-NGS-ONP180821NB19/2021	EPI_ISL_3746811	Free State	Unknown	Tree, temporal analysis, highlighter plot
hCoV-19/SouthAfrica/UFS-VIRO-NGS-ONP180821NB28/2021	EPI_ISL_3746772	Free State	Unknown	Tree, temporal analysis, highlighter plot
hCoV-19/SouthAfrica/UFS-VIRO-NGS-ONP180821NB45/2021	EPI_ISL_3746842	Free State	Unknown	Tree, temporal analysis, highlighter plot
hCoV-19/SouthAfrica/UFS-VIRO-NGS-ONP180821NB47/2021	EPI_ISL_3746871	Free State	Unknown	Highlighter plot

hCoV-19/SouthAfrica/UFS-VIRO-NGS-ONP180821NB50/2021	EPI_ISL_3746752	Free State	Unknown	Tree, temporal analysis, highlighter plot
hCoV-19/SouthAfrica/UFS-VIRO-NGS-ONP180821NB53/2021	EPI_ISL_3746788	Free State	Unknown	Tree, temporal analysis, highlighter plot
hCoV-19/SouthAfrica/UFS-VIRO-NGS-ONP180821NB71/2021	EPI_ISL_3746874	Free State	Unknown	Highlighter plot
hCoV-19/SouthAfrica/VIDA-KRISP-K018954/2021	EPI_ISL_2841668	Gauteng	Unknown	Tree, temporal analysis, highlighter plot
hCoV-19/SouthAfrica/VIDA-KRISP-K018963/2021	EPI_ISL_2841677	Gauteng	Unknown	Tree, temporal analysis, highlighter plot
hCoV-19/SouthAfrica/VIDA-KRISP-K020434/2021	EPI_ISL_3729072	Gauteng	Yes	Tree, temporal analysis, highlighter plot
hCoV-19/SouthAfrica/VIDA-KRISP-K020445/2021	EPI_ISL_3729227	Gauteng	Yes	Tree, temporal analysis, highlighter plot

Supplementary Table 2: Reference set of C.1.2 genomes on GISAID from other countries. We gratefully acknowledge the following authors from the originating laboratories responsible for obtaining the specimen, as well as the submitting laboratories where the genomes were generated and shared via GISAID, on which this research is based. All submitters of data may be contacted via www.gisaid.org. Provided are the GISAID strain name and GISAID_EPI_ISL accession numbers, authors (listed according to how they were provided on GISAID), travel status and use in various analyses for non-South African C.1.2 samples deposited into GISAID as of September, 10, 2021.

Strain Name	GISAID Accession ID	Country	Travel History	Use in Analysis	Originating Laboratory	Submitting Laboratory	Author List
hCoV-19/Botswana/R23B38_BHP_000667728/2021	EPI_ISL_3453877	Botswana	Unknown	Tree, temporal analysis, highlighter plot	Palapye Primary Hospital Laboratory	Botswana Harvard HIV Reference Laboratory	Sikhulile Moyo, Wonderful T. Choga, Dorcas Maruapula, Thongbotho Mphoyakgosi, Boitumelo J.L Zuze, Botshelo Radibe, Legodile Kooepile, Ontlametse T. Bareng, Letsibogo Gaoraelwe, Thela Tefelo, Keoratile Ntshambiwa, Modisa Motswaledi, Madisa Mine, Joseph Makhema, Roger Shapiro, Shahin Lockman, Mosepele Mosepele, Simani Gaseitsiwe
hCoV-19/Shenzhen/IVDC-0610-33/2021	EPI_ISL_2931281	China	Yes	Tree, temporal analysis, highlighter plot	Shenzhen Center for Disease Control and Prevention	National Institute for Viral Disease Control and Prevention, China CDC	Long Chen, Can Zhu,Xinyi Wei,Renli Zhang,Kai Nie, Peihua Niu,Weihua Wu,Yue Li,Shaoyu Deng and Yaqing He
hCoV-19/DRC/INRB-RDC-557/2021	EPI_ISL_3086931	Democratic Republic of the Congo	Unknown	Highlighter plot	Viral Respiratory Lab, National Institute for Biomedical Research (INRB)	Pathogen Sequencing Lab, National Institute for Biomedical Research (INRB)	Placide Mbala-Kingebeni, Edith Nkwembe, Eddy Kinganda-Lusamaki, Amuri Aziza, Francisca Muyembe Mawete, Emmanuel Lokilo Lofiko, Jean Claude Makangara, Raphaël Lumembe, Gabriel Kabamba, Catherine Pratt, Matthias Pauthner, Josh Quick, Allison Black, James Hadfield, Trevor Bedford, Ian Goodfellow, Andrew Rambaut, Nick Loman, Kristian Andersen, Michael Wiley, Steve Ahuka-Mundeke, Jean-Jacques Muyembe Tamfum
hCoV-19/Eswatini/NICD-N13384/2021	EPI_ISL_4301774	Eswatini	Unknown	Tree, temporal analysis, highlighter plot	National Reference Laboratory (NRL), Eswatini	National Institute for Communicable Diseases of the National Health Laboratory Service	Maphalala G, Amoako DG, Everatt J, Scheepers C, Mohale T, Ntuli N, Mahlangu B, Mnguni A, Ismail A, Bhiman JN
hCoV-19/Eswatini/NICD-N13401/2021	EPI_ISL_4301791	Eswatini	Unknown	Tree, temporal analysis, highlighter plot	National Reference Laboratory (NRL), Eswatini	National Institute for Communicable Diseases of the National Health Laboratory Service	Maphalala G, Amoako DG, Everatt J, Scheepers C, Mohale T, Ntuli N, Mahlangu B, Mnguni A, Ismail A, Bhiman JN
hCoV-19/Eswatini/NICD-N13438/2021	EPI_ISL_4301822	Eswatini	Yes	Tree, temporal analysis, highlighter plot	National Reference Laboratory (NRL), Eswatini	National Institute for Communicable Diseases of the National Health Laboratory Service	Maphalala G, Amoako DG, Everatt J, Scheepers C, Mohale T, Ntuli N, Mahlangu B, Mnguni A, Ismail A, Bhiman JN
hCoV-19/Eswatini/NICD-N13445/2021	EPI_ISL_4301828	Eswatini	Yes	Tree, temporal analysis, highlighter plot	National Reference Laboratory (NRL), Eswatini	National Institute for Communicable Diseases of the National Health Laboratory Service	Maphalala G, Amoako DG, Everatt J, Scheepers C, Mohale T, Ntuli N, Mahlangu B, Mnguni A, Ismail A, Bhiman JN
hCoV-19/Eswatini/NICD-N13453/2021	EPI_ISL_4301836	Eswatini	Unknown	Tree, temporal analysis, highlighter plot	National Reference Laboratory (NRL), Eswatini	National Institute for Communicable Diseases of the National Health Laboratory Service	Maphalala G, Amoako DG, Everatt J, Scheepers C, Mohale T, Ntuli N, Mahlangu B, Mnguni A, Ismail A, Bhiman JN
hCoV-19/Eswatini/NICD-N13454/2021	EPI_ISL_4301837	Eswatini	Yes	Highlighter plot	National Reference Laboratory (NRL), Eswatini	National Institute for Communicable Diseases of the National Health Laboratory Service	Maphalala G, Amoako DG, Everatt J, Scheepers C, Mohale T, Ntuli N, Mahlangu B, Mnguni A, Ismail A, Bhiman JN

hCoV-19/Eswatini/NICD-N13372/2021	EPI_ISL_4301764	Eswatini	Yes	Tree, temporal analysis, highlighter plot	National Reference Laboratory (NRL), Eswatini	National Institute for Communicable Diseases of the National Health Laboratory Service	Maphalala G, Amoako DG, Everatt J, Scheepers C, Mohale T, Ntuli N, Mahlangu B, Mnguni A, Ismail A, Bhiman JN
hCoV-19/Portugal/PT11580/2021	EPI_ISL_2989113	Portugal	Unknown	Tree, temporal analysis, highlighter plot	SESARAM	Instituto Nacional de Saude (INSA)	Borges et al
hCoV-19/NewZealand/21MV0551/2021	EPI_ISL_3164100	New Zealand	Yes	Tree, temporal analysis, highlighter plot	Middlemore Hospital	Institute of Environmental Science and Research (ESR)	Rachel Boyle, SallyAnn Harbison, Olivia Stroeven, Xiaoyun Ren, Matt Storey, Nikki Freed, Muhammad Faisal, Jing Wang, Hermes Perez, Anja Werno, Antje van der Linden, Arlo Upton, Chris Mansell, David Hammer, Dragana Drinkovic, Gary McAuliffe, Hana Sofia Andersson, James Ussher, Jill Sherwood, Josh Freeman, Julia Howard, Juliet Elvy, Mary DeAlmeida, Matt Blakiston, Matthew Rogers, Max Bloomfield, Michael Addidle, Michelle Balm, Sally Roberts, Sarah Jefferies, Sharmini Muttaiyah, Susan Morpeth, Susan Taylor, Timothy Blackmore, Vani Sathyendran, Veronica Playle, Virginia Hope, Erasmus Smit, Lauren Jelly, Olin Silander, Joep de Ligt
hCoV-19/Mauritius/235422/2021	EPI_ISL_3236186	Mauritius	Yes	Tree, temporal analysis, highlighter plot	Airport Health Laboratory/Central Health Laboratory	Virology Department, Central Health Laboratory, Victoria Hospital, Candos,Ministry of Health and Wellness, Mauritius	Manraj SS, Sonoo J, Pattoo M, Bahadoor BS, Mathur H, Sujeewon C, Jannoo N, Ramuth M
hCoV-19/England/MILK-176835E/2021	EPI_ISL_2718062	United Kingdom	Yes	Tree, temporal analysis, highlighter plot	Lighthouse Lab in Milton Keynes	Wellcome Sanger Institute for the COVID-19 Genomics UK (COG-UK) Consortium	The Lighthouse Lab in Milton Keynes and Alex Alderton, Roberto Amato, Jeffrey Barrett, Sonia Goncalves, Ewan Harrison, David K. Jackson, Ian Johnston, Dominic Kwiatkowski, Cordelia Langford, John Sillitoe on behalf of the Wellcome Sanger Institute COVID-19 Surveillance Team
hCoV-19/England/MILK-17E1CDA/2021	EPI_ISL_2803815	United Kingdom	Yes	Tree, temporal analysis, highlighter plot	Lighthouse Lab in Milton Keynes	Wellcome Sanger Institute for the COVID-19 Genomics UK (COG-UK) Consortium	The Lighthouse Lab in Milton Keynes and Alex Alderton, Roberto Amato, Jeffrey Barrett, Sonia Goncalves, Ewan Harrison, David K. Jackson, Ian Johnston, Dominic Kwiatkowski, Cordelia Langford, John Sillitoe on behalf of the Wellcome Sanger Institute COVID-19 Surveillance Team
hCoV-19/England/MILK-1A58D6F/2021	EPI_ISL_3287712	United Kingdom	Yes	Tree, temporal analysis, highlighter plot	Lighthouse Lab in Milton Keynes	Wellcome Sanger Institute for the COVID-19 Genomics UK (COG-UK) Consortium	The Lighthouse Lab in Milton Keynes and Alex Alderton, Roberto Amato, Jeffrey Barrett, Sonia Goncalves, Ewan Harrison, David K. Jackson, Ian Johnston, Dominic Kwiatkowski, Cordelia Langford, John Sillitoe on behalf of the Wellcome Sanger Institute COVID-19 Surveillance Team
hCoV-19/England/MILK-1C2C773/2021	EPI_ISL_3697115	United Kingdom	Yes	Tree, temporal analysis, highlighter plot	Lighthouse Lab in Milton Keynes	Wellcome Sanger Institute for the COVID-19 Genomics UK (COG-UK) Consortium	The Lighthouse Lab in Milton Keynes and Alex Alderton, Roberto Amato, Jeffrey Barrett, Sonia Goncalves, Ewan Harrison, David K. Jackson, Ian Johnston, Dominic Kwiatkowski, Cordelia Langford, John Sillitoe on behalf of the Wellcome Sanger Institute COVID-19 Surveillance Team
hCoV-19/Switzerland/VD-CHUV-GEN5512/2021	EPI_ISL_2868597	Switzerland	Unknown	Highlighter plot	Établissements Hospitaliers du Nord Vaudois (EHNV)	Laboratory of genomics and metagenomics	Trestan Pillonel, Damien Jacot, Sébastien Aeby, Gilbert Greub, Claire Bertelli
hCoV-19/Switzerland/ZH-UZH-IMV-3ba4c99a/2021	EPI_ISL_3128775	Switzerland	Yes	Tree, temporal analysis, highlighter plot	Stadtspital Triemli	Institute of Medical Virology	Daniel Ehrsam, Isabel Stürmer, Catharine Aquino, Joel Wirz, Weihong Qi, Hubert Rehrauer, Verena Kufner, Gabriela Ziltener, Maryam Zaheri, Stefan Schmutz, Annette Audigé, Maria Grünberg, Kevin Steiner, Jon Huder, Cyril Shah, Riccarda Capaul, Guido Bloemberg, Jürg Böni, Michael Huber, Alexandra Trkola

hCoV-19/Zimbabwe/CERI-KRISP-K021327/2021	EPI_ISL_3722284	Zimbabwe	No	Tree, temporal analysis, highlighter plot	National Microbiology Reference Laboratory, Ministry of Health, Harare, Zimbabwe	CERI, Centre for Epidemic Response and Innovation, Stellenbosch University and KRISP, KZN Research Innovation and Sequencing Platform, UKZN	Air Comodor Dr J. Chimedza, Dr Raiva Simbi, Agnes Juru, Tapfumanei Mashe, Kenneth Maeka, Hlanai Gumbo, Tatenda Takawira, Charles Nyagupe, Giandhari J, Pillay S, Naidoo Y, Emmanuel SJ, Tegally H, Wilkinson E, de Oliveira T

Supplementary Table 3: Characteristics of the SARS-CoV-2 convalescent study participants for the pseudovirus neutralization assays

Cohort ID	Cohort	Variant/strain by infection date	Wave	Age Range (y)	Sex	Tested against			
						D614G	Beta	C.1.2	Delta
COV004	Steve Biko Hospital	D614G	1	60 – 69	M	Yes	Yes	Yes	Yes
COV006	Steve Biko Hospital	D614G	1	20 – 29	M	Yes	Yes	Yes	Yes
COV020	Steve Biko Hospital	D614G	1	50 – 59	M	Yes	No	Yes	No
COV021	Steve Biko Hospital	D614G	1	40 – 49	F	Yes	No	No	No
COV024	Steve Biko Hospital	D614G	1	60 – 69	M	Yes	No	No	No
COV025	Steve Biko Hospital	D614G	1	40 – 49	F	Yes	Yes	Yes	Yes
COV036	Steve Biko Hospital	D614G	1	40 – 49	M	Yes	Yes	Yes	Yes
COV042	Steve Biko Hospital	D614G	1	30 – 39	M	Yes	Yes	Yes	Yes
COV043	Steve Biko Hospital	D614G	1	50 – 59	M	Yes	No	Yes	No
COV044	Steve Biko Hospital	D614G	1	40 – 49	M	Yes	No	No	No
SA-01-0025	Groote Schuur Hospital	Beta	2	50 – 59	F	Yes	Yes	Yes	Yes
SA-01-0032	Groote Schuur Hospital	Beta	2	60 – 69	F	Yes	Yes	Yes	Yes
SA-01-0042	Groote Schuur Hospital	Beta	2	40 – 49	F	Yes	Yes	No	Yes
SA-01-0052	Groote Schuur Hospital	Beta	2	60 – 69	F	Yes	Yes	Yes	Yes
SA-01-0081	Groote Schuur Hospital	Beta	2	60 – 69	F	Yes	Yes	No	Yes
SA-01-0018	Groote Schuur Hospital	Beta	2	60 – 69	M	Yes	Yes	Yes	Yes
SA-01-0038	Groote Schuur Hospital	Beta	2	40 – 49	M	Yes	Yes	Yes	Yes
SA-01-0040	Groote Schuur Hospital	Beta	2	50 – 49	M	Yes	Yes	Yes	Yes
SA-01-0068	Groote Schuur Hospital	Beta	2	30 – 39	M	Yes	Yes	Yes	Yes
SA-01-0075	Groote Schuur Hospital	Beta	2	70+	M	Yes	Yes	No	Yes
COV101	Steve Biko Hospital	Delta	3	40 – 49	M	Yes	Yes	Yes	Yes
COV105	Steve Biko Hospital	Delta	3	60 – 69	M	Yes	Yes	Yes	Yes
COV108	Steve Biko Hospital	Delta	3	50 – 59	M	Yes	Yes	Yes	Yes
COV110	Steve Biko Hospital	Delta	3	40 – 49	M	Yes	Yes	Yes	Yes
COV113	Steve Biko Hospital	Delta	3	60 – 69	F	Yes	Yes	Yes	Yes
COV114	Steve Biko Hospital	Delta	3	60 – 69	M	Yes	Yes	No	Yes
COV116	Steve Biko Hospital	Delta	3	60 – 69	F	Yes	Yes	Yes	Yes
COV124	Steve Biko Hospital	Delta	3	40 – 49	F	Yes	Yes	Yes	Yes
COV125	Steve Biko Hospital	Delta	3	70+	M	Yes	Yes	No	Yes

Supplementary Table 4: Characteristics of the vaccine study participants for the pseudovirus neutralization assays

Cohort ID	Vaccine	Age (y)	Sex	Days post-full vaccination	Tested against			
					D614G	Beta	C.1.2	Delta
1-10036	AZD1222 (2 shots)	20 – 29	F	14	Yes	Yes	Yes	Yes
1-10037	AZD1222 (2 shots)	40 – 49	F	14	Yes	Yes	Yes	Yes
1-10043	AZD1222 (2 shots)	30 – 39	M	14	Yes	Yes	Yes	Yes
1-10044	AZD1222 (2 shots)	10 – 19	F	14	Yes	Yes	Yes	Yes
1-10020	AZD1222 (2 shots)	30 – 39	M	14	Yes	Yes	Yes	Yes
1-10048	AZD1222 (2 shots)	50 – 59	F	14	Yes	Yes	Yes	Yes
1-10058	AZD1222 (2 shots)	10 – 19	F	14	Yes	Yes	Yes	Yes
1-10019	AZD1222 (2 shots)	10 – 19	M	14	Yes	Yes	Yes	Yes
1-10023	AZD1222 (2 shots)	20 – 29	M	14	Yes	Yes	Yes	Yes
1-10026	AZD1222 (2 shots)	40 – 49	M	14	Yes	Yes	Yes	Yes
1-10011	AZD1222 (2 shots)	30 – 39	M	14	Yes	Yes	Yes	Yes
945	Ad26.COV2.S (1 shot)	30 – 39	F	62	Yes	Yes	Yes	Yes
946	Ad26.COV2.S (1 shot)	30 – 39	F	62	Yes	Yes	Yes	Yes
948	Ad26.COV2.S (1 shot)	50 – 59	F	62	Yes	Yes	Yes	Yes
949	Ad26.COV2.S (1 shot)	20 – 29	F	62	Yes	Yes	Yes	Yes
950	Ad26.COV2.S (1 shot)	30 – 39	F	62	Yes	Yes	Yes	Yes
951	Ad26.COV2.S (1 shot)	30 – 39	F	62	Yes	Yes	Yes	Yes
952	Ad26.COV2.S (1 shot)	20 – 29	F	62	Yes	Yes	No	Yes
953	Ad26.COV2.S (1 shot)	30 – 39	F	62	Yes	Yes	Yes	Yes
955	Ad26.COV2.S (1 shot)	30 – 39	F	62	Yes	Yes	Yes	Yes
956	Ad26.COV2.S (1 shot)	20 – 29	F	62	Yes	Yes	Yes	Yes
930	BNT162b2 (2 shots)	60 – 69	F	62	Yes	Yes	Yes	Yes
931	BNT162b2 (2 shots)	60 – 69	F	62	Yes	Yes	Yes	Yes
936	BNT162b2 (2 shots)	70+	F	62	Yes	Yes	Yes	Yes
933	BNT162b2 (2 shots)	70+	F	62	Yes	Yes	Yes	Yes
928	BNT162b2 (2 shots)	70+	M	62	Yes	Yes	Yes	Yes
929	BNT162b2 (2 shots)	70+	F	62	Yes	Yes	Yes	Yes
932	BNT162b2 (2 shots)	60 – 69	M	62	No	No	Yes	No

Supplementary Table 5: Characteristics of the SARS-CoV-2 convalescent study participants for the live virus neutralization assay.

Cohort ID	Variant/strain by infection date	Variant/strain by sequencing	Wave	Sequence ID	GISAID Accession	Age range (y)	Sex	Days between symptom onset and plasma collection
039-02-0031	Beta	Beta	2	K008636	N/A*	40 – 49	F	41
039-02-0033	Beta	Beta	2	K008637	EPI_ISL_1229368	50 – 59	M	42
039-02-0034	Beta	N/A[$]	2	N/A[$]	N/A	30 – 39	F	32
039-02-0046	Beta	Beta	2	K010372	N/A*	30 – 39	M	33
039-02-1011	Beta	Beta	2	K010356	N/A*	70+	F	48
039-09-0001	Beta	Beta	2	K008633	EPI_ISL_1229367	60 – 69	F	29
039-02-0045	Beta	Beta	2	K010370	N/A*	30 – 39	F	31
039-02-0104	Delta	Delta	3	K021407	EPI_ISL_3722338	40 – 49	F	26
039-02-0106	Delta	Delta	3	K021399	EPI_ISL_3722335	40 – 49	M	23[#]
039-02-0108	Delta	Delta	3	K021401	N/A*	50 – 59	M	31
039-02-0109	Delta	Delta	3	K021225	N/A*	40 – 49	M	13[#]
039-13-0153	Delta	Delta	3	K021400	N/A*	40 – 49	M	44
039-13-0140	Delta	Delta	3	K021226	N/A*	50 – 59	M	44
039-13-0141	Delta	Delta	3	K020186	EPI_ISL_3939068	40 – 49	M	31
039-13-0142	Delta	Delta	3	K020187	EPI_ISL_3939088	30 – 39	M	31
039-13-0149	Delta	Delta	3	K020214	EPI_ISL_3447779	50 – 59	F	30[#]

[*] <90% coverage. Not submitted to GISAID but sufficient sequence for variant call. [$] Not sequenced. [#]Asymptomatic at diagnosis, date post-diagnostic test used instead of symptom onset date.

Supplementary Table 6: Characteristics of the Pfizer BNT162b2 vaccinated participants for the live virus neutralization assay.

Cohort ID	Age range (y)	Sex	Days post-second dose
136070	20 – 29	M	87
136072	30 – 39	F	134
136074	20 – 29	M	131
136075	50 – 59	M	152
136076	30 – 39	F	153
136078	30 – 39	M	158
