# Supplementary material for: Emergence and phenotypic characterization of the global SARS-CoV-2 C.1.2 lineage
Source: Nat Commun. 2022 Apr 8;13:1976. doi: 10.1038/s41467-022-29579-9 (PMC8993834; doi:10.1038/s41467-022-29579-9)
Supplement: Supplementary file 3 — Description of Additional Supplementary Files [file 41467_2022_29579_MOESM3_ESM.pdf]

Description of Additional Supplementary Files

File Name: Supplementary Software 1
Description: C.1.2 code .zip containing necessary datasets and custom scripts (in addition a GitHub link is available for all scripts used at https://github.com/NICD-CRDM/C.1.2_scripts).
