# Supplementary material for: Emergence and phenotypic characterization of the global SARS-CoV-2 C.1.2 lineage
Source: Nat Commun. 2022 Apr 8;13:1976. doi: 10.1038/s41467-022-29579-9 (PMC8993834; doi:10.1038/s41467-022-29579-9)
Supplement: Supplementary file 5 — Reporting Summary [file 41467_2022_29579_MOESM5_ESM.pdf]

nature portfolio

Corresponding author(s): Jinal Bhiman

Last updated by author(s): 19 Jan 2022

Reporting Summary

Nature Portfolio wishes to improve the reproducibility of the work that we publish. This form provides structure for consistency and transparency in reporting. For further information on Nature Portfolio policies, see our Editorial Policies and the Editorial Policy Checklist.

Statistics

For all statistical analyses, confirm that the following items are present in the figure legend, table legend, main text, or Methods section.

n/a	Confirmed	
☐	☒	The exact sample size (*n*) for each experimental group/condition, given as a discrete number and unit of measurement
☐	☒	A statement on whether measurements were taken from distinct samples or whether the same sample was measured repeatedly
☐	☒	The statistical test(s) used AND whether they are one- or two-sided<br>*Only common tests should be described solely by name; describe more complex techniques in the Methods section.*
☒	☐	A description of all covariates tested
☒	☐	A description of any assumptions or corrections, such as tests of normality and adjustment for multiple comparisons
☐	☒	A full description of the statistical parameters including central tendency (e.g. means) or other basic estimates (e.g. regression coefficient) AND variation (e.g. standard deviation) or associated estimates of uncertainty (e.g. confidence intervals)
☐	☒	For null hypothesis testing, the test statistic (e.g. *F*, *t*, *r*) with confidence intervals, effect sizes, degrees of freedom and *P* value noted<br>*Give P values as exact values whenever suitable.*
☐	☒	For Bayesian analysis, information on the choice of priors and Markov chain Monte Carlo settings
☒	☐	For hierarchical and complex designs, identification of the appropriate level for tests and full reporting of outcomes
☒	☐	Estimates of effect sizes (e.g. Cohen's *d*, Pearson's *r*), indicating how they were calculated

Our web collection on statistics for biologists contains articles on many of the points above.

Software and code

Policy information about availability of computer code

Data collection	SARS-CoV-2 genome and metadata was collected from GISAID (www.epicov.org) using their download tool specific for the augur pipeline. Data was collected based on submissions up to and including September 10, 2021.
Data analysis	Genomes from Illumina reads were assembled using either the Exatype NGS SARS-CoV-2 pipeline v1.6.1 with consensus sequences cleaned using Aliview v1.27, or Genome Detective v.1.132/1.133 and the Coronavirus Typing Tool. Genomes from nanopore reads were assembled according to the Artic-nCoV2019 novel coronavirus bioinformatics protocol or using the Fastq QC + ARTIC + NextClade pipeline on Epi2Me (Oxford Nanopore Technologies), with initial assemblies validated using Geneious v2021.0.3, and consensus sequences cleaned using Aliview v1.27. Phylogenetic analysis was performed using Nextstrain (https://github.com/nextstrain/ncov, v8, accessed 29 August 2021). Molecular clock analysis was performed using IQ-TREE v.1.6.9 and TempEst v.1.5.3. Phylogeographic analysis was performed using BEAST v1.10.4 and Tracer v.1.7.1, as well as the R package seraphim. Recombination analysis was performed using RDP5 (http://web.cbio.uct.ac.za/~darren/rdp.html).

For manuscripts utilizing custom algorithms or software that are central to the research but not yet described in published literature, software must be made available to editors and reviewers. We strongly encourage code deposition in a community repository (e.g. GitHub). See the Nature Portfolio guidelines for submitting code & software for further information.

Data

Policy information about availability of data

All manuscripts must include a data availability statement. This statement should provide the following information, where applicable:

- Accession codes, unique identifiers, or web links for publicly available datasets
- A description of any restrictions on data availability
- For clinical datasets or third party data, please ensure that the statement adheres to our policy

All of the global SARS-CoV-2 genomes generated and presented in this article are publicly accessible through the GISAID platform (https://www.gisaid.org/), along with all other SARS-CoV-2 genomes generated by the NGS-SA. The GISAID accession identifiers of the C.1.2 sequences analyzed in this study are provided as part of Supplementary Tables 1 and 2, which also contain the metadata for the sequences. The Nextstrain build of C.1.2 and global sequences is available at https://nextstrain.org/groups/ngs-sa/COVID19-C.1.2-2022-01-05. The GISAID accession identifiers for the full set of sequences used in this build can be accessed at https://github.com/NICD-CRDM/C.1.2_scripts/Nextstrain_files. The GISAID accession identifiers for the sequences used in Supp. Fig. 2a and temporal analysis can be accessed at https://github.com/NICD-CRDM/C.1.2_scripts.

Field-specific reporting

Please select the one below that is the best fit for your research. If you are not sure, read the appropriate sections before making your selection.

☒ Life sciences ☐ Behavioural & social sciences ☐ Ecological, evolutionary & environmental sciences

For a reference copy of the document with all sections, see nature.com/documents/nr-reporting-summary-flat.pdf

Life sciences study design

All studies must disclose on these points even when the disclosure is negative.

Sample size	For genomic analysis, due to the size of the GISAID SARS-CoV-2 sequence repository (containing 3.7 million genomes), a sub-sampling was performed to obtain a manageable representation of genomes. For C.1.2 this included all genomes with more than 90% sequence coverage (n=156). For non-C.1.2 lineages, 6,030 genomes were selected for the custom Nextstrain build used in this manuscript, which included 1,850 genomes from South Africa, 1,039 from other African countries, 901 from Asia, 1,126 from Europe, 484 from South America, 430 from North America and 205 from Oceania.<br>For functional assay data, no sample size calculation was performed, but rather plasma specimens were selected to include low, medium and high neutralizing titre or ADCC activity against the D614G virus. Between 5-10 plasma samples from previously infected individuals were used. Between 6-15 plasma samples from vaccinated individuals were used. Samples were selected based on availability and unique biological material was analysed.
Data exclusions	C.1.2 genomes were only excluded if they had less than 90% genome coverage.
Replication	Genome sequencing results were generated once and were not repeated. For functional assays, each plasma samples was tested in duplicate and only successful results were reported. IC50 and ADCC signaling titres were averaged, data is shown as geometric means and error bars indicate 95% confidence intervals for all samples/virus.
Randomization	Matched samples were selected based on neutralization titres against D614G, representing a range of neutralization (low, medium and high).
Blinding	Blinding was not relevant to this study as it did not include a clinical trial or trial-related investigations. Blinding was not needed as there was no chance for conscious or unconscious bias that could have affected the outcome of the functional assays.

Reporting for specific materials, systems and methods

We require information from authors about some types of materials, experimental systems and methods used in many studies. Here, indicate whether each material, system or method listed is relevant to your study. If you are not sure if a list item applies to your research, read the appropriate section before selecting a response.

Materials & experimental systems

n/a	Involved in the study	
☐	☒	Antibodies
☐	☒	Eukaryotic cell lines
☒	☐	Palaeontology and archaeology
☒	☐	Animals and other organisms
☒	☐	Human research participants
☒	☐	Clinical data
☒	☐	Dual use research of concern

Methods

n/a	Involved in the study	
☒	☐	ChIP-seq
☒	☐	Flow cytometry
☒	☐	MRI-based neuroimaging

Antibodies

Antibodies used	anti-IgG APC (clone QA19A42) - Biolegend (Cat#366905; RRID:AB_2888847)<br>CR3022 (Genscript (https://www.genscript.com)<br>P2B-2F6 (Dr Nicole Doria-Rose, VRC, NIH, USA)<br>Palivizumab (Medimmune ; Synagis; RRID: AB_2459638)
Validation	CR0322, P2B-2F6 and Palivizumab have previously been validated (https://www.cell.com/cell-reports-medicine/fulltext/S2666-3791(22)00040-4). Anti-IgG APC is commercially available and has been appropriately validated for specific species or application by the manufacturer and provided in their datasheets as follows: https://www.biolegend.com/en-us/products/apc-anti-human-igg-fc-recombinant-antibody-20025?pdf=true&displayInline=true&leftRightMargin=15&topBottomMargin=15&filename=APC%20anti-human%20IgG%20Fc%20Recombinant%20Antibody.pdf

Eukaryotic cell lines

Policy information about cell lines

Cell line source(s)	293T/ACE2.MF, 293T/17, ACE2-expressing H1299-E3, Vero E6, HEK293T, Jurkat-Lucia NFAT-CD16 cells
Authentication	None of the cell lines were authenticated
Mycoplasma contamination	All cell lines tested negative for mycolplasma
Commonly misidentified lines<br>(See ICLAC register)	None used in this study
